# Layers of inhibitory networks shape receptive field properties of AII amacrine cells

**DOI:** 10.1016/j.celrep.2023.113390

**Published:** 2023-11-05

**Authors:** Amurta Nath, William N. Grimes, Jeffrey S. Diamond

**Affiliations:** 1 Synaptic Physiology Section, National Institute of Neurological Disorders and Stroke, National Institutes of Health, Bethesda, MD 20892, USA; 2 Lead contact

## Abstract

In the retina, rod and cone pathways mediate visual signals over a billion-fold range in luminance. AII (“A-two”) amacrine cells (ACs) receive signals from both pathways via different bipolar cells, enabling AIIs to operate at night and during the day. Previous work has examined luminance-dependent changes in AII gap junction connectivity, but less is known about how surrounding circuitry shapes AII receptive fields across light levels. Here, we report that moderate contrast stimuli elicit surround inhibition in AIIs under all but the dimmest visual conditions, due to actions of horizontal cells and at least two ACs that inhibit presynaptic bipolar cells. Under photopic (daylight) conditions, surround inhibition transforms AII response kinetics, which are inherited by downstream ganglion cells. Ablating neuronal nitric oxide synthase type-1 (nNOS-1) ACs removes AII surround inhibition under mesopic (dusk/dawn), but not photopic, conditions. Our findings demonstrate how multiple layers of neural circuitry interact to encode signals across a wide physiological range.

## In brief

Nath et al. show that receptive fields of AII (“A-two”) amacrine cells (ACs) are shaped by strong surround suppression under mesopic and photopic conditions. Ablating nNOS1 ACs removes mesopic surround only. Changes in AII surround suppression are inherited by downstream RGCs, thereby influencing the retinal output.

## Graphical Abstract

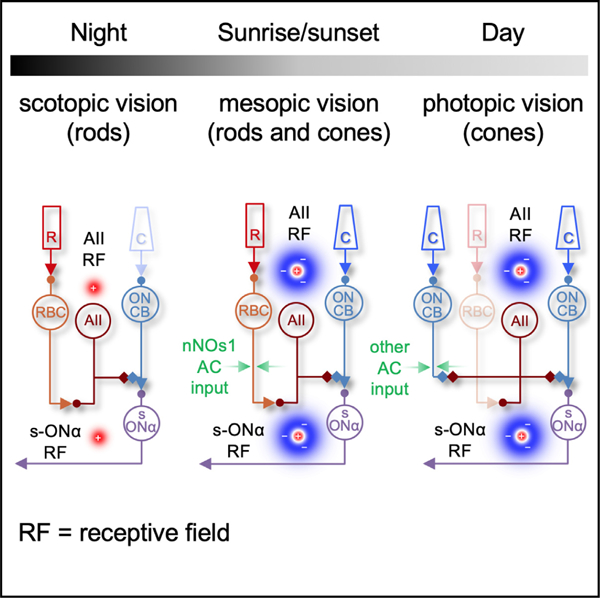

## INTRODUCTION

Interactions between excitatory and inhibitory signaling underlie neural computations throughout the nervous system.^1^ This is clearly manifested in a ubiquitous property of sensory processing, the antagonistic center-surround organization of receptive fields (RFs). The RF center in retinal ganglion cells (RGCs) arises from excitatory inputs from bipolar cells,^2,3^ whereas the inhibitory surround is formed by horizontal cells (HCs) in the outer retina and amacrine cells (ACs) in the inner retina.^4–7^ This RF organization enables RGCs to transmit contrast, color, edge, and spatial and temporal frequency information based on the relative activity in the RF center and surround. As the statistics of the visual environment change from night to day, RF dimensions^8^ and the relative size of the center and surround^9^ can change accordingly. Several studies have reported weak or absent surrounds in dark-adapted states,^8,10,11^ whereas others have observed antagonistic surrounds in dim light.^9,12^ Most studies have focused on RGCs; less is known about how the visual environment influences AC RFs.

**Figure 1.**
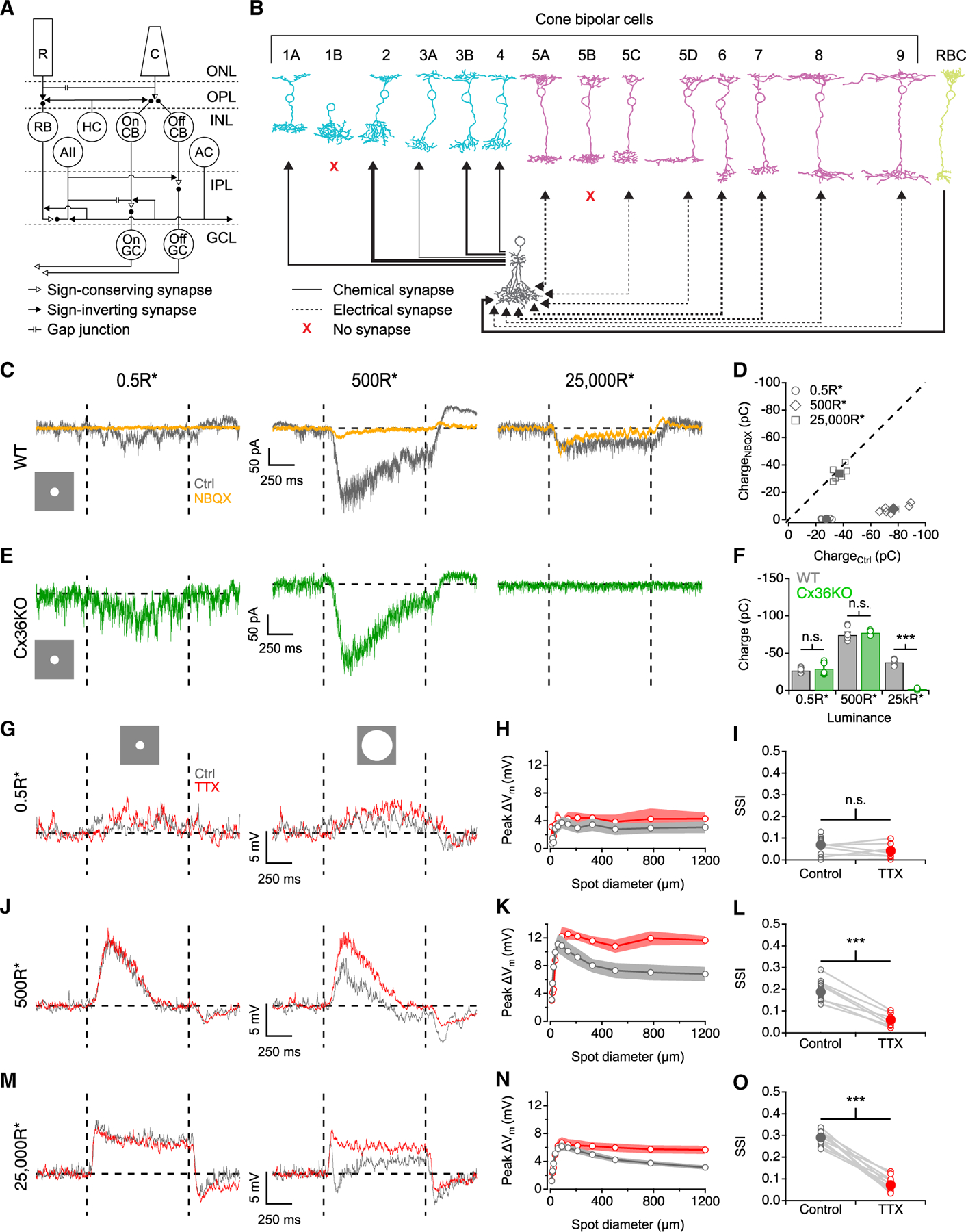
Surround suppression of AII ACs changes with luminance (A) Neuronal circuitry underlying rod and cone vision in mouse retina. R, rod photoreceptor; C, cone photoreceptor; RB, rod bipolar cell; CB, cone bipolar cell; HC, horizontal cell; AC, amacrine cell; GC, ganglion cell; ONL, outer nuclear layer; OPL, outer plexiform layer; INL, inner nuclear layer; IPL, inner plexiform layer; GCL, ganglion cell layer. Diagram adapted from Vaney et al.^79^ (B) Synaptic connections between bipolar cell types and AII ACs. Weights of lines indicate number of connections between AII and bipolar cell type on a log_10_ scale. Data are taken from Tsukamoto and Omi.^28^ Bipolar cell drawings were adapted from Shekhar et al.^80^ (C) AII EPSCs evoked by an 88 μm spot (+100% Weber contrast) from a background of 0.5 (left), 500 (middle), and 25,000 R*/rod/s (right). Vertical dashed lines indicate beginning and end of light stimulus. Horizontal dashed line indicates average AII membrane potential before stimulus. (D) Summary of charge during stimulus interval in response to an 88 μm spot in control vs. NBQX conditions. (E) AII EPSCs evoked by an 88 μm spot at 0.5 (left), 500 (middle) and 25,000 R*/rod/s (right) in Cx36KO retina. (F) Summary of charge during stimulus interval for an 88 μm spot in WT and Cx36KO retina at different backgrounds (open circles, individual cells; n = 6 for WT and n = 5 for Cx36KO for each luminance). (G) AII membrane responses to an 88 μm spot (left) and 1,200 μm spot (right) from a 0.5 R*/rod/s background (+100% Weber contrast). (H) AII membrane depolarization vs. spot diameter at 0.5 R*/rod/s background (n = 13 for control and n = 7 for TTX). (I) SSI plotted in control and TTX conditions across the cell population (open circles, individual cells; closed circles, population mean; n = 13 for control and n = 7 for TTX). (J–L) As in (G)–(I) but for 500 R*/rod/s (n = 13 for control and n = 7 for TTX). (M–O) As in (G)–(I) but for 25,000 R*/rod/s (n = 12 for control and TTX). Data are represented as mean ± SEM. ***p < 0.001.

At night, rod photoreceptors^13,14^ transmit single-photon signals to rod bipolar cells (RBCs),^15,16^ which convey ON signals to AIIs (“A-two”). AIIs relay rod signals to the cone pathway via electrical and chemical synapses onto ON and OFF cone bipolar cells (CBCs), respectively^17–19^ (Figure 1A). During daytime, AIIs convey ON CBC signals, traveling in the opposite direction through those same electrical synapses, to RGCs.^20–22^ AIIs are coupled to each other via electrical synapses^23,24^; coupling strength among AIIs in rabbit retina varies with background luminance.^25,26^ This suggests that AII RF center size might vary with increasing light levels in other species^27^ and that gap junctions shape AII RFs. Since AIIs are connected to most bipolar cell types (Figure 1B) via chemical or electrical synapses,^28^ changes in AII RF properties may dramatically influence signaling in the inner retina and, consequently, the retinal output.

Here, we recorded light responses from AIIs in mouse retina and measured AII RF properties at various light levels. We show that AIIs exhibit ON sustained visual responses and very little surround suppression under scotopic conditions. As background luminance increases to mesopic levels, wide-field ACs (WACs) deliver increasing surround inhibition directly onto AIIs and to presynaptic RBC terminals. Surround suppression negatively rectifies AII responses to flickering stimuli and, under photopic conditions, dramatically changes response time course. These kinetic changes affect retinal output via ON CBCs, as reflected in sustained ON alpha (s-ONα) RGC light responses. HCs also contribute to presynaptic surround suppression under mesopic and photopic conditions. Viral ablation of neuronal nitric oxide synthase type-1 (nNOS-1) ACs reduced surround suppression only in the mesopic range and linearized AII responses to flickering light. Together, our findings suggest that multiple retinal circuits influence AII RFs, which shape retinal output.

## RESULTS

### AIIs receive distinct excitatory inputs at different light levels

AIIs in wild-type (WT) mouse retinas were targeted for patch recordings in the whole-mount preparation based on soma shape and proximity to the inner nuclear layer (INL) and inner plexiform layer (IPL) border. Our first goal was to identify the circuit pathways by which excitatory visual signals reach AIIs under our different luminance conditions. AIIs receive convergent input from RBCs via AMPA-type glutamate receptors^29,30^ and from ON CBCs via Connexin36 (Cx36)-containing gap junctions.^31–33^ RBC-mediated inputs can therefore be blocked selectively by the AMPAR antagonist NBQX, effectively isolating input from the ON CBC (NBQX-insensitive) pathway.^21,34,35^ Photoreceptor signals to ON bipolar cell dendrites are transmitted by mGluR6 receptors and therefore are unaffected by NBQX.^36,37^ To understand how relative contributions of these inputs change over light levels, we voltage clamped AII ACs at the reversal potential for inhibition (~ −60 mV) and recorded excitatory postsynaptic currents (EPSCs) evoked by small spots of light (88 μm, +100% Weber contrast) comparable in size to AII RF centers.^38^ At 0.5 R*/rod/s background, NBQX eliminated the light responses (97.7% ± 1.4% charge reduction, n = 6), indicating that these scotopic signals are transmitted primarily through RBCs, with negligible contribution from CBCs (Figures 1C, left, and 1D). At 500 R*/rod/s (mesopic levels), EPSCs were strongly diminished by NBQX (89.8% ± 2.9% charge reduction, n = 6), indicating that most input came from RBCs, with only a minor CBC component (Figures 1C, middle, and 1D). At photopic levels (25,000 R*/rod/s), NBQX had minimal effect on AII EPSCs (7.7% ± 12.3% charge reduction, n = 6; Figures 1C and 1D), suggesting that CBCs provide photopic input to AIIs with little contribution from RBCs. These results were corroborated by experiments conducted in retinas from mice in which *cjd2*, the gene encoding Cx36, was knocked out (referred to henceforth as Cx36KO), thereby eliminating electrical synapses between AIIs and ON CBCs.^31^ Light responses of comparable magnitudes to WT retinas for spots of same contrast were recorded in Cx36KO retinas under scotopic and mesopic conditions (p = 0.64 and 0.84, unpaired t test, respectively; Figures 1E and 1F). EPSCs were absent in Cx36KO AIIs at 25,000 R*/rod/s (Figures 1E and 1F), confirming that photopic signals reach AIIs through the ON CBC pathway.

OFF CBCs also provide ribbon inputs to AIIs,^28,39^ and OFF-center responses have been recorded from AIIs in rabbit retina in the presence of _L_-AP4, an mGluR6 agonist.^40^ We did not observe any OFF responses in AIIs in control conditions,^39^ suggesting that OFF inputs to AIIs may be inhibited by the ON pathway.

### AII RFs exhibit a luminance-dependent inhibitory surround

We next examined how AII light-evoked postsynaptic potentials (PSPs) and RFs change across different luminance levels. AIIs were depolarized in response to positive contrast (+100%) spots, first under scotopic conditions (Figure 1G, left). We defined RF center size by the smallest stimulus spot that produced maximal AII depolarization (100.9 ± 13.6 μm, n = 13). AII RF centers were substantially larger than the lateral extent of AII dendritic arbors (~30 μm),^38^ likely reflecting gap-junctional coupling within the AII^23,24,33^ and AII-ON CBC networks.^18,32,33^ Similar depolarizations were recorded for larger spots exceeding RF center dimensions (Figures 1G and 1H), indicating a lack of surround suppression under scotopic conditions. Spot stimuli delivered at 500 R*/rod/s revealed larger AII responses (peak ΔV = 3.7 ± 0.8 vs. 10.9 ± 1.4 mV in scotopic vs. mesopic conditions, respectively [88 μm spot]). The measured RF center was 96.4 ± 9.4 μm (n = 13) in mesopic conditions, similar to scotopic measurements (p = 0.79, unpaired t test). Under photopic conditions, RF center size (92.4 ± 7.1 μm, n = 12) remained statistically indistinguishable from that measured at lower light levels (p = 0.6 and 0.75, unpaired t test vs. scotopic and mesopic, respectively). The similarity of RF center size across luminance contradicts previous studies in rabbit retina reporting changes in AII center size with increase in background illumination.^26,27^ This discrepancy could reflect species-specific differences and/or aspects of our whole-mount retina preparation that minimized gap-junctional modulation (see discussion), enabling us to examine other circuit features that influence AII RFs.

Spots larger than the AII RF center elicited robust surround suppression in both mesopic and photopic conditions (Figures 1J–1O). To quantify the degree of surround suppression, we compared responses to small and large spot sizes with a surround suppression index (SSI; Figure 1I; see STAR Methods) ranging between 0 (no surround suppression) and 1 (complete surround suppression). SSI values were minimal at scotopic backgrounds (mean SSI = 0.07 ± 0.01, n = 13) and increased at mesopic (mean SSI = 0.19 ± 0.01, n = 13, p = 3.5 × 10^−7^, unpaired t test) and photopic (mean SSI = 0.29 ± 0.01, n = 12, p = 7.7 × 10^−14^, unpaired t test) backgrounds.

Tetrodotoxin (TTX) blocks voltage-gated sodium channels and suppresses output from WACs and surround inhibition in AIIs.^40–42^ Accordingly, bath-applied TTX (500 nM) reduced surround suppression under mesopic and photopic conditions (Figures 1J–1O), reflected in lower SSI values (p = 5.6 × 10^−6^ and 1.34 × 10^−8^ for mesopic and photopic, respectively). No significant change in scotopic SSI was observed after TTX application at 0.5 R*/rod/s (p = 0.11, unpaired t test).

### Surround suppression observed in AIIs has pre- and postsynaptic components

**Figure 2.**
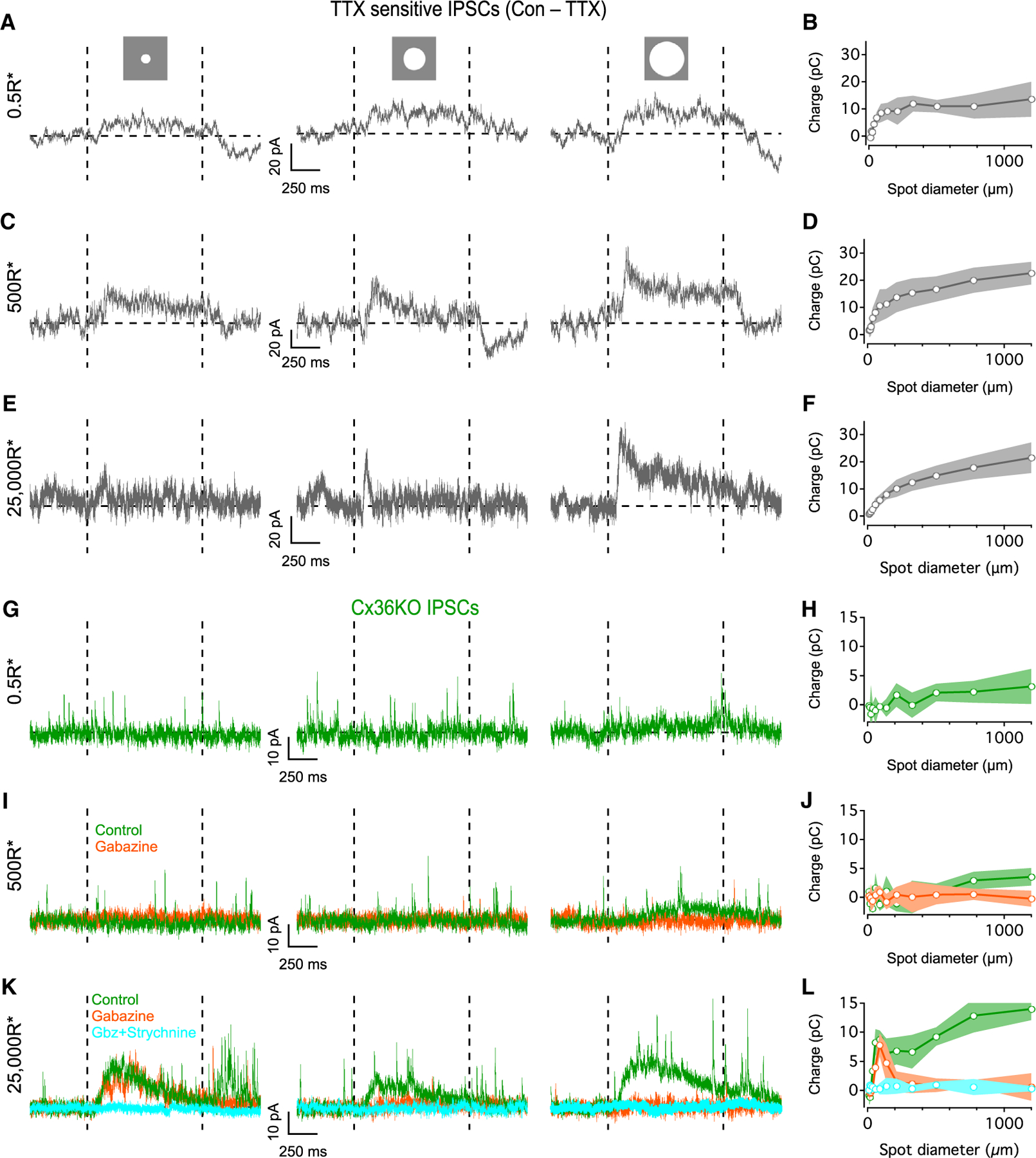
AII IPSCs at different backgrounds (A) TTX-sensitive IPSCs evoked in AIIs by an 88 μm spot (left), 325 μm spot (middle), and 1,200 μm spot (right) from a 0.5 R*/rod/s background (+100% Weber contrast). (B) Charge during stimulus interval vs. spot diameter at 0.5 R*/rod/s background (n = 8). (C and D) As in (A) and (B) but for 500 R*/rod/s background (n = 8). (E and F) As in (A) and (B) but for 25,000 R*/rod/s background (n = 8). (G) AII IPSCs recorded in Cx36KO retina to an 88 μm spot (left), 325 μm spot (middle), and 1,200 μm spot (right) from a 0.5 R*/rod/s background (+100% Weber contrast). (H) As in (B) but in Cx36KO (n = 5). (I) As in (G) but in Cx36KO. (J) As in (B) but in Cx36KO (n = 5). (K and L) As in (E) and (F) but in Cx36KO (n = 5). Data are represented as mean ± SEM.

We next determined whether surround suppression in AII RFs is due to presynaptic or postsynaptic inhibition, as both AIIs and presynaptic bipolar cells receive TTX-sensitive inhibitory input from WACs.^42,43^ Postsynaptic inhibition is typically measured by clamping the membrane potential near the excitatory reversal potential (E_cation_ ~0 mV) and recording inhibitory postsynaptic currents (IPSCs). In AIIs, this approach is complicated by extensive Cx36-mediated electrical coupling among AIIs, causing IPSCs to be contaminated by unclamped gap-junctional currents.^42^ Residual inward coupling currents remained in TTX, which abolished the inhibitory conductance (Figure S1).^42^ TTX-sensitive IPSCs were isolated by subtracting the currents from two different conditions (control — TTX; Figures 2A–2F). IPSCs increased in magnitude as a function of spot diameter for all light levels, as expected from a wide-field source of inhibition. The inhibitory charge for the largest spot size (1,200 μm) was larger in brighter mean illumination than scotopic conditions (mean charges = 13.6 ± 6.5, 22.5 ± 4.2, and 21.5 ± 5.7 pC in scotopic, mesopic, and photopic respectively, n = 8 each; p = 0.002 and 0.02 for scotopic vs. mesopic and scotopic vs. photopic, respectively, unpaired t tests).

To achieve better space clamp, we also recorded IPSCs in Cx36KO retinas in which AIIs are electrically isolated.^31^ Smaller IPSCs were recorded for similar stimuli at all light levels compared to WT (Figures 2G–2L), likely owing to reduced sensitivity in Cx36KO retinas.^36^ IPSCs evoked by large spots (400–1,200 μm) were blocked completely by the GABA_A_ antagonist gabazine (Figures 2I–2L). Interestingly, under photopic conditions, we observed narrow-field inhibition (Figure 2K, left, peak charge = 7.8 ± 2.0 pC at 88 μm, n = 5) that was resistant to gabazine and blocked by the glycine receptor antagonist strychnine (Figures 2K and 2L). This glycinergic input also appeared subject to surround suppression, as it was absent in responses to larger (>200 μm) spot sizes. These experiments demonstrate that direct inhibition to AIIs comprises GABAergic and glycinergic components that exhibit distinct spatial profiles.

**Figure 3.**
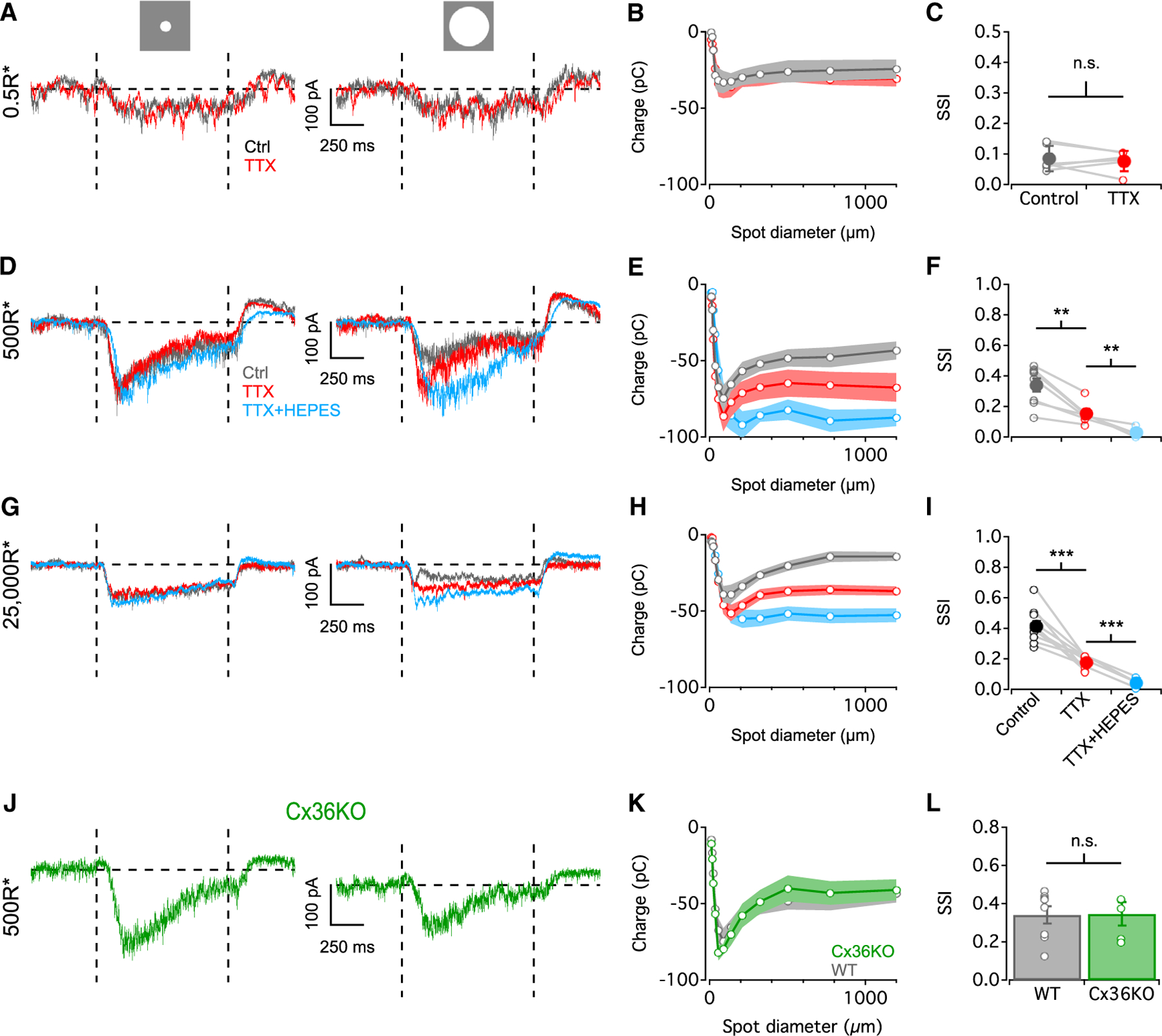
AII EPSCs at different backgrounds (A) AII EPSCs evoked by an 88 μm spot (left) and 1,200 μm spot (right) from a 0.5 R*/rod/s background (+100% Weber contrast). (B) Charge during stimulus interval vs. spot diameter at 0.5 R*/rod/s background (n = 6). (C) SSI plotted in control and TTX conditions across the cell population (open circles, individual cells; closed circles, population mean; n = 6). (D) As in (A) but for 500 R*/rod/s background. (E) As in (B) but for 500 R*/rod/s background (n = 8 for control and TTX, n = 5 for TTX+HEPES). (F) SSI plotted in control, TTX, and TTX+HEPES across the cell population (n = 8 for control and TTX, n = 5 for TTX+HEPES). (G–I) As in (D)–(F) but for 25,000 R*/rod/s background (n = 10 for control and TTX, n = 6 for TTX+HEPES). (J) AII EPSCs recorded in Cx36KO retina to an 88 μm spot (left) and 1,200 μm spot (right) from a background of 500 R*/rod/s (+100% Weber contrast). (K) As in (E) but in Cx36KO (n = 5) and WT (n = 8). (L) SSI plotted in WT and Cx36KO mice across the cell population (open circles, individual cells; n = 8 for WT and n = 5 for Cx36KO). Data are represented as mean ± SEM. **p < 0.01, ***p < 0.001.

To examine the influence of presynaptic inhibition on excitatory inputs to AIIs, we recorded EPSCs (Figure 3). Under scotopic conditions, EPSCs exhibited little surround suppression (mean SSI = 0.08 ± 0.02; Figures 3A–3C) and remained unaltered when TTX was added to the bath solution, with no significant changes in SSI (p = 0.62, paired t test, mean SSI = 0.07 ± 0.01; Figures 3A–3C). Strong surround suppression of EPSCs was observed at higher background light levels (SSI = 0.34 ± 0.04 and 0.41 ± 0.03 in mesopic and photopic conditions, respectively; Figures 3D–3I) and was reduced by TTX (mean SSIs = 0.15 ± 0.02 and 0.17 ± 0.01 in mesopic and photopic conditions, respectively, p = 0.0008 and 0.0001, paired t test; Figures 3D–3I).

Some presynaptic surround persisted in the presence of TTX at mesopic and photopic levels (Figures 3E and 3H), suggesting that a component of surround suppression in AIIs is mediated by non-spiking ACs or some other source. HCs mediate lateral inhibition in the outer retina^44^ via a complex mechanism^45^ that can be blocked by buffering extracellular pH with HEPES.^46,47^ Applying HEPES (20 mM) together with TTX eliminated surround suppression in EPSCs (mean SSIs = 0.04 ± 0.02 and 0.04 ± 0.01 in mesopic and photopic, respectively; Figures 3D–3I), indicating that HCs influence AII RFs. HC signaling can be modulated via TTX-dependent mechanisms,^48–50^ possibly causing us to underestimate the degree of HC contribution to AII surround. Applying HEPES prior to TTX did not significantly change mesopic or photopic surround suppression (p = 0.52 and 0.07 for control vs. HEPES at mesopic and photopic, respectively, paired t test; Figure S2), however, indicating that TTX did not occlude a larger HC effect and that HCs contribute a relatively minor component of the presynaptic surround under our experimental conditions.

Non-spiking, GABAergic A17 ACs inhibit RBC terminals^51^ and might thereby contribute to AII presynaptic surround inhibition. We ruled out this possibility, however, because (1) surround inhibition was eliminated upon application of TTX and HEPES (Figures 3D–3I) and (2) A17 neuritic arbors are ~400 μm in diameter^52^ and so probably cannot mediate surround suppression across the larger spatial scales tested here.

To test the influence of gap junctions on presynaptic surround inhibition, we recorded AII EPSCs in Cx36 KO retinas. Surround inhibition persisted at mesopic levels in Cx36KO (Figures 3J and 3K), and SSIs were not significantly different compared to WT (p = 0.84, unpaired t test; Figure 3L). Since AIIs use only Cx36 to form gap junctions,^23,33^ this suggests that AII gap junctions do not contribute significantly to surround suppression. By a similar logic, these results suggest that Cx36-mediated gap junctions between photoreceptors^53^ do not contribute to surround suppression either. EPSCs were absent under photopic conditions in Cx36KO AIIs (Figures 1E and 1F), preventing us from evaluating the contribution of electrical synapses.

**Figure 4.**
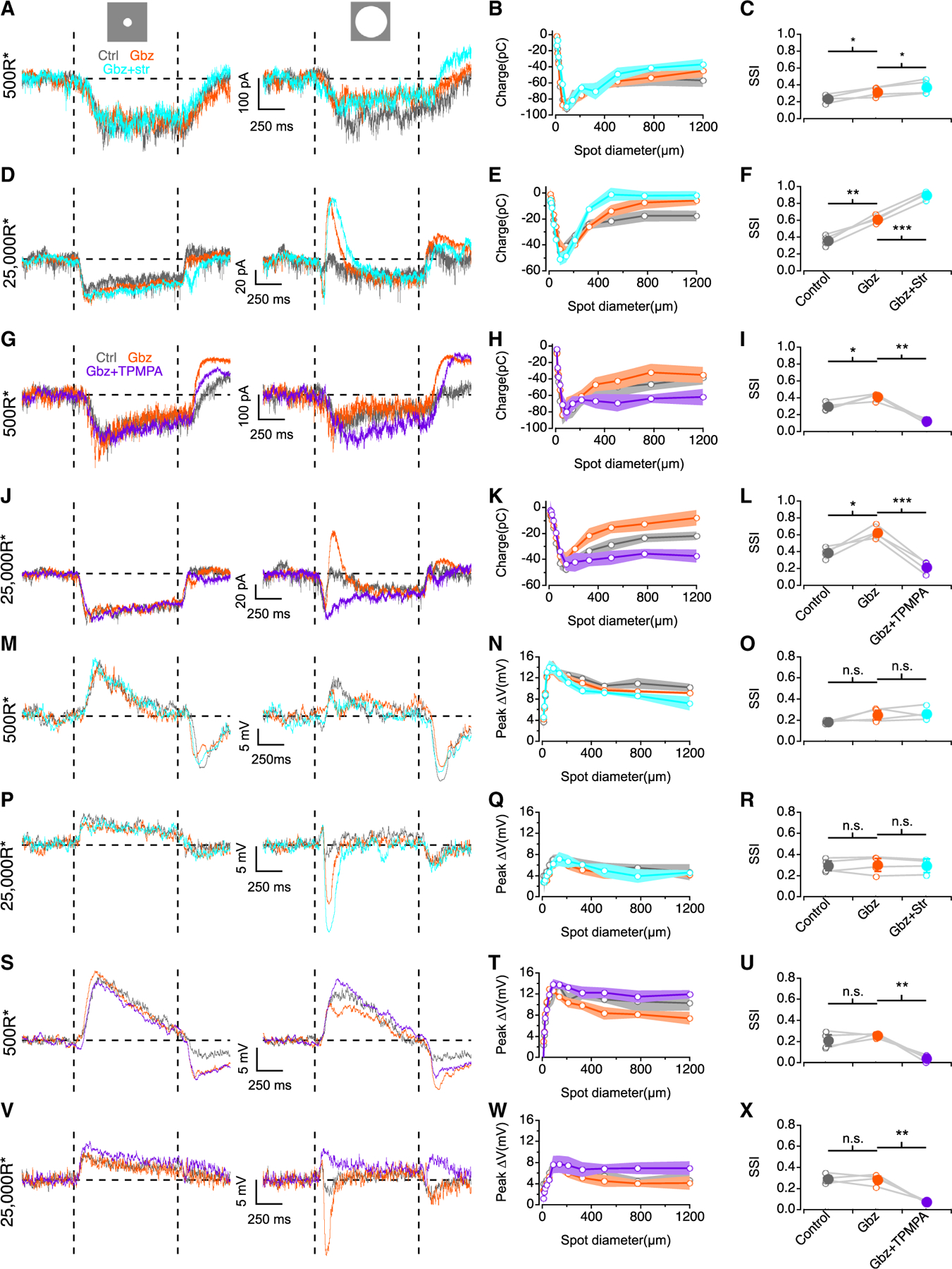
Surround suppression is presynaptic and mediated by GABA_C_ receptors (A) AII EPSCs in response to an 88 μm spot (left) and 1,200 μm spot (right) from a 500 R*/rod/s background (+100% Weber contrast). (B) Charge during stimulus interval vs. spot diameter at 500 R*/rod/s background (n = 4). (C) SSI plotted in control, gabazine, and gabazine+strychnine conditions across the cell population (open circles, individual cells; closed circles, population mean; n = 4). (D–F) As in (A)–(C) but for 25,000 R*/rod/s background (n = 4). (G and I) As in (A)–(C) but with gabazine and gabazine+TPMPA (n = 4). (J–L) As in (D)–(F) but with gabazine and gabazine+TPMPA (n = 4). (M) As in (A) but for AII PSPs. (N) AII membrane depolarization vs. spot diameter at 500 R*/rod/s background (n = 4). (O) As in (C) but for AII PSPs (n = 4). (P–R) As in (M)–(O) but for 25,000 R*/rod/s background (n = 4). (S–U) As in (G)–(I) but for AII PSPs (n = 4). (V–X) As in (S)–(U) but for 25,000 R*/rod/s background (n = 4). Data are represented as mean ± SEM. *p < 0.05, **p < 0.01, ***p < 0.001.

We next examined which synaptic receptors mediate presynaptic surround inhibition (Figure 4). Blocking GABA_A_ and glycine receptors actually enhanced surround suppression of EPSCs at mesopic and photopic levels (p = 0.04 and 0.02 for control vs. gabazine at 500R* and 25kR*, respectively, p = 0.002 and 0.0007 for gabazine vs. gabazine+strychnine at 500R* and 25kR*, respectively, paired t tests; Figures 4A–4F). TPMPA, a GABA_C_ receptor antagonist, greatly reduced surround suppression (Figure 4G–4L), lowering SSIs significantly at both light levels (p = 0.002 and 0.0007 for gabazine vs. gabazine+TPMPA at mesopic and photopic, respectively, paired t tests; Figure 4I and 4L). GABA_C_ receptors are localized primarily at bipolar cell presynaptic terminals,^54–56^ suggesting that bipolar cells providing input to AIIs receive surround suppression, largely mediated by GABA_C_ receptors, under mesopic and photopic conditions. Furthermore, these results suggest that serial inhibition by other GABAergic and glycinergic ACs,^57^ acting via GABA_A_ and glycine receptors, regulates this presynaptic inhibition. Light-evoked AII PSPs exhibited similar behavior: gabazine and strychnine boosted surround suppression at mesopic and photopic levels (Figures 4M–4R), whereas TPMPA reduced it (Figures 4S–4X), indicating that GABA_c_Rs influence spatial aspects of AII RFs.

### Surround suppression rectifies AII responses

**Figure 5.**
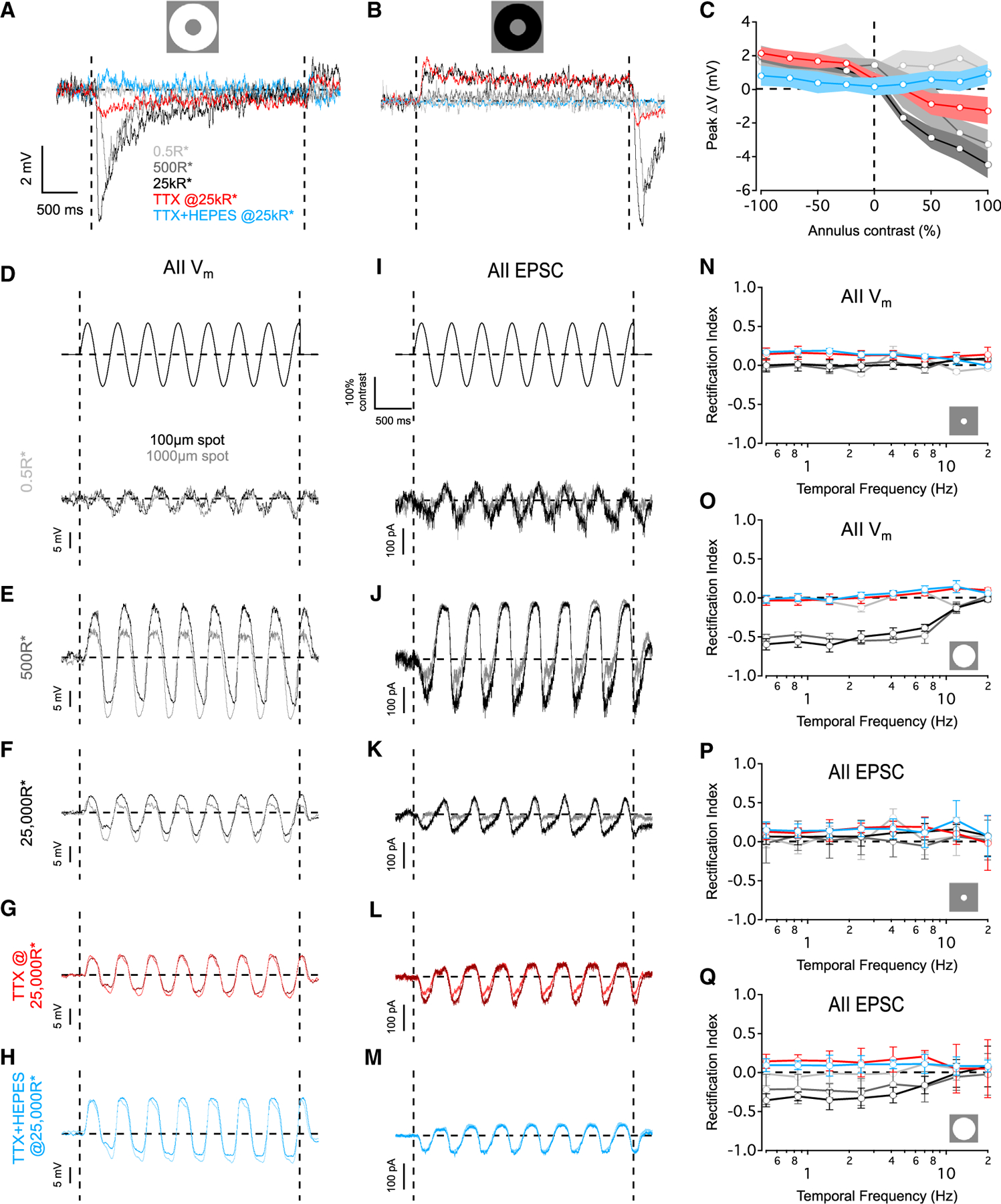
Surround suppression rectifies AII output (A) AII PSPs evoked by an annulus (+100% Weber contrast) at different backgrounds. (B) As in (A) but for −100% Weber contrast. (C) Summary of AII PSPs vs. annulus contrast (n = 10 cells for 0.5, 500, and 25,000 R*/rod/s and TTX, n = 8 cells for TTX+HEPES). (D) AII PSPs (bottom row) evoked by sinusoidally modulated contrast (top row). Responses to 100 μm spot (black) and 1,000 μm spot (dark gray) are plotted together for a 2.43Hz temporally modulated stimulus presented from a 0.5 R*/rod/s background. (E–H) Same as in (D) but for backgrounds of 500 (E) and 25,000R*/rod/s (F), TTX at 25,000 R*/rod/s background (G), and TTX+HEPES at 25,000 R*/rod/s background (H). (I–M) As in (D)–(H) but for AII current responses voltage clamped at E_Cl_. (N–O) Rectification index of AII membrane responses plotted vs. temporal frequency (n = 8 for 0.5, 500, and 25,000 R*/rod/s and TTX, n = 5 cells for TTX+HEPES). Color scheme as in (A). (P and Q) Rectification index of AII EPSCs plotted vs. temporal frequency (n = 8 for 0.5, 500, and 25,000 R*/rod/s and TTX, n = 5 cells for TTX+HEPES). Data are represented as mean ± SEM. Color scheme as in (A).

In the experiments presented thus far, spot stimuli activated the center and the surround concurrently. Next, to examine interactions between surround inhibition and center excitation, we first activated the surround alone with an annular stimuli (250 μm inner diameter, 1,000 μm outer diameter) at varying contrasts under different background luminance conditions (Figure 5; see STAR Methods). Annuli presented atop a 0.5 R*/rod/s background did not elicit any responses (Figures 5A–5C), consistent with a lack of surround suppression under our scotopic stimulus conditions (Figure 1). Mesopic annular stimuli elicited a transient hyperpolarization (peak ΔV = −3.3 ± 0.9 mV, n = 10) that decreased to a sustained level in response to positive contrasts (Figure 5A). Under photopic conditions, AII responses to positive contrasts were similar in kinetics and became slightly larger in magnitude (peak ΔV = −4.5 ± 0.8 mV, n = 10). TTX eliminated the transient component of the annular response (Figures 5A and 5C); a residual, sustained component was blocked by HEPES, indicating an HC contribution (Figures 5A and 5C). Negative contrast annuli elicited sustained responses that were entirely blocked by HEPES (Figures 5B and 5C). These results suggest that transient surround suppression is mediated by ON-driven spiking ACs.

Transient surround inhibition might influence responses to continuously changing visual stimuli more than it affects responses to the sustained (step) stimuli that we have delivered thus far. To test this idea, we recorded AII PSPs evoked by 100 and 1,000 μm spots presented at contrasts that were sinusoidally modulated at various frequencies atop different background levels. Robust responses were recorded for both spot sizes at all light levels for frequencies <8 Hz (Figures S3D and S3E). Under scotopic conditions, spots of both sizes elicited similar responses (Figure 5D). The effect of surround suppression was revealed for large spot sizes under mesopic and photopic conditions: AIIs responded with a large hyperpolarization to the OFF phase and smaller depolarization to the ON phase of the stimulus (Figures 5E and 5F). AIIs responded linearly to narrow-field time-varying stimuli, i.e., with ON depolarizations and OFF hyperpolarizations of equal magnitude (Figures 5E and 5F), components that were distinctly evident in responses to the onset and offset of conventional small-spot stimuli (e.g., Figures 1 and 4). We quantified these observations with a rectification index (see STAR Methods). This index ranges from −1 (negatively rectified), to 0 (non-rectified), to 1 (positively rectified). No rectification was measured in scotopic responses to either spot size (Figures 5D, 5N, and 5O). Compared to scotopic conditions, both mesopic and photopic responses to large stimuli were negatively rectified (p = 0.0005, one-way ANOVA, F = 11.05, degrees of freedom [DF] = 23) whereas responses to small spots were not (p = 0.91, one-way ANOVA, F = 0.09, DF = 23; Figures 5N and 5O). Negative rectification to large spots was eliminated by TTX (p = 0.0001, paired t test), and little change was observed after further perfusion of HEPES (p = 0.13, paired t test; Figures 5G, 5H, and 5O). Note that both surround inhibition and rectification were absent in scotopic responses, emerged in mesopic and photopic responses, and were blocked by TTX (cf. Figures 1, 3, and 5O), suggesting that surround inhibition rectifies a linear visual signal provided by the excitatory center in response to temporally modulated stimuli.

Most surround suppression appears due to presynaptic inputs (Figures 3 and 4), so we tested whether activation of the surround rectifies AII EPSCs. As expected, responses to the annuli were absent at 0.5 R*/rod/s (Figures S3A–S3C). At mesopic and photopic levels, AIIs exhibited transient and sustained components in response to positive contrasts (Figures S3A and S3C). Similar to our observations for AII PSPs, the transient component was blocked by TTX, the sustained component was blocked by subsequent application of HEPES, and the transient component was absent in responses to negative contrast annuli (Figures S3A–S3C). As expected, AII EPSCs were linear for scotopic sinusoidally modulated stimuli (Figures 5I, 5P, and 5Q). Negative rectification of AII EPSCs emerged for large sinusoidally modulated spots at mesopic and photopic levels and was abolished by TTX (Figures 5J–5L, 5P, and 5Q; p = 0.002, paired t test). Further application of HEPES had no significant effect (p = 0.07, paired t test; Figures 5M and 5Q). These results suggest that AIIs mostly transmit information about low temporal frequencies (<8 Hz) and that surround suppression negatively rectifies mesopic and photopic signals. Changes in signal rectification, commonly observed in retinal circuitry, may drive a shift from linear to non-linear spatial integration.^34^

### Ablating nNOS-1 ACs removes AII surround in mesopic conditions

**Figure 6.**
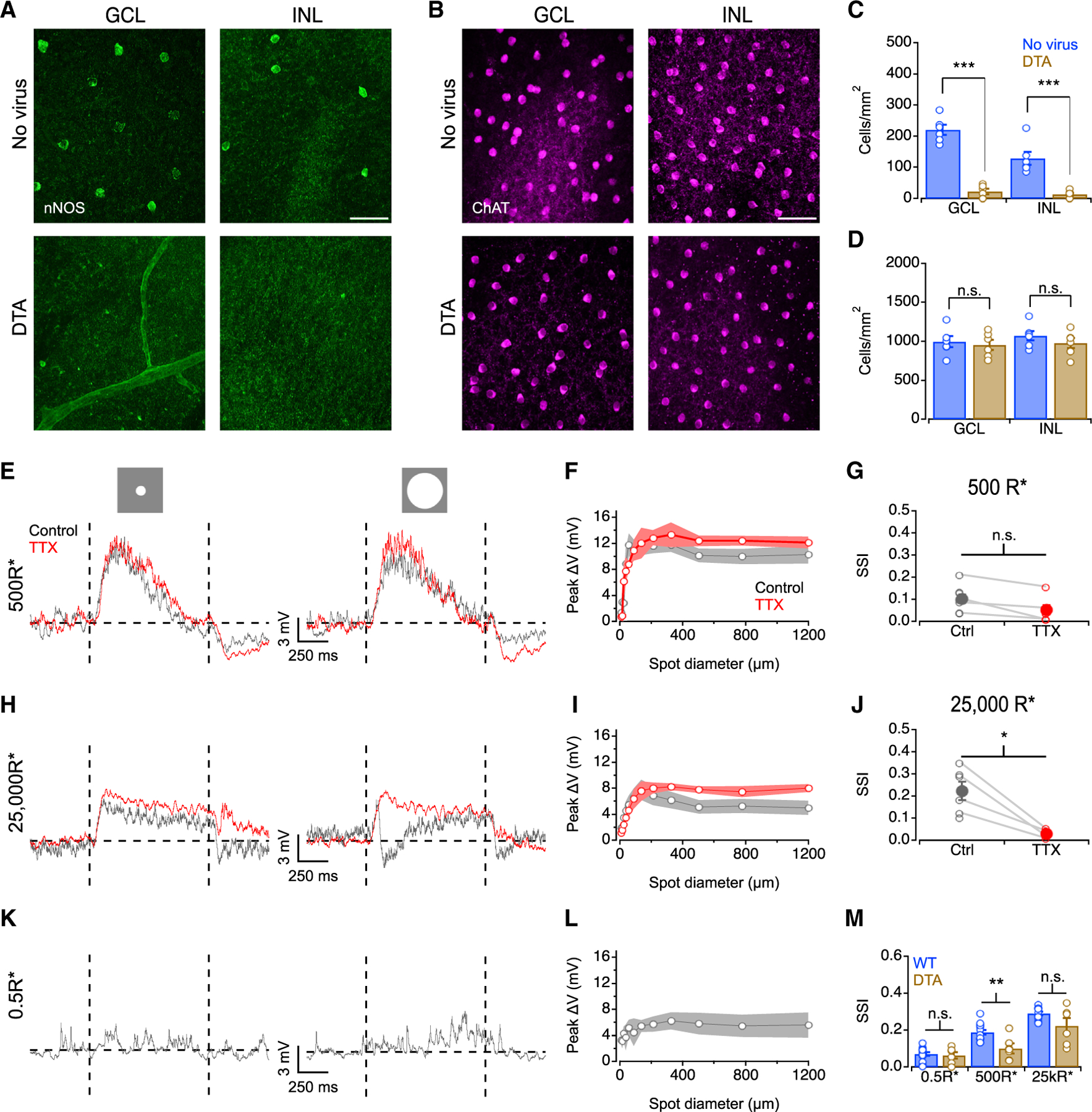
Ablating nNOS1 ACs changes AII surround properties (A) nNOS immunolabeling in GCL and INL. Scale bar: 50 μm. (B) ChAT immunolabeling in GCL and INL. Scale bar: 50 μm. (C) Density of nNOS+ somas calculated over a square region (224.91 × 224.91 μm). Open circles represent individual cells. Error bars indicate ± SEM across cells (n = 6 for both WT and DTA ablated retina). (D) Same as in (L) but for ChAT^+^ starburst AC somas. (E) AII PSPs in a nNOS-CreER/tdTomato (TdTom) retina post-DTA ablation to an 88 μm spot (left) and a 1,200 μm spot (right) from a 0.5 R*/rod/s background (+100% Weber contrast). (F) AII membrane depolarization vs. spot diameter at 500 R*/rod/s background (n = 8 for control and n = 5 for TTX). (G) SSI plotted in control and TTX at 500 R*/rod/s background across the cell population (open circles, individual cells; closed circles, population mean; n = 8 for control and n = 5 for TTX). (H–J) As in (E)–(G) but for 25,000R*/rod/s background (n = 6 for control and n = 4 for TTX). (K and L) As in (E) and (F) but for 0.5R*/rod/s background (n = 6). (M) SSI plotted in WT and DTA ablated nNOS-CreER/TdTom retina across the cell population. Open circles represent individual cells (WT n as in Figure 1, DTA n as above). Data are represented as mean ± SEM. *p < 0.05, **p < 0.01, ***p < 0.001.

The nNOS-1 AC, an ON wide-field spiking interneuron, provides most of its synaptic output to AIIs and RBCs and contributes to surround inhibition of AIIs evoked by dim stimuli presented atop a completely dark background.^42^ We hypothesized that nNOS-1 ACs might also mediate surround inhibition at higher light levels. To test this, we ablated these cells in nNOS-CreER transgenic mice^58^ by intraocularly injecting a virus expressing diphtheria toxin (DTA) in a Cre-dependent manner^42^ (see STAR Methods). The efficacy of DTA ablation was verified post hoc via nNOS antibody staining. nNOS immunopositive somas were reduced significantly following DTA injection (88.7% ± 10.3% and 89% ± 9.3% reduction in the ganglion cell layer (GCL) and the INL, respectively; Figures 6A and 6C). ChAT-positive starburst AC numbers in virally infected retinas were similar to those in WT (p = 0.71 and 0.32 for GCL and INL, respectively, unpaired t test; Figures 6B and 6D), indicating that DTA expression exerted few off-target effects.^42^

Four weeks following injection, AII membrane voltages were recorded in response to spots of increasing diameters. Mesopic responses were similar to WT for small spots, but response amplitudes were not diminished for increasing spot sizes (Figures 6E–6G), a significant difference from the strong mesopic surround observed in WT (p = 0.004, unpaired t test; Figure 6M), suggesting that nNOS-1 ACs provide surround suppression to AIIs under mesopic conditions. Accordingly, TTX exerted no significant effects on the surround at this light level (Figures 6E–6G), confirming that DTA ablation of nNOS ACs effectively removed mesopic surround suppression. Under photopic conditions, however, strong, TTX-sensitive surround suppression persisted, comparable to WT (p = 0.14, unpaired t test; Figures 6H–6J and 6M), and AII response time courses remained complex for large spots. Scotopic responses were similar to those in WT retina and lacked surround suppression (p = 0.66, unpaired t test; Figures 6K–6M). These results suggest that a different GABAergic AC provides surround presynaptic inhibition to AIIs under photopic conditions.

When AIIs were probed with small (100 μm) and large (1,000 μm) spots sinusoidally modulated at scotopic levels following DTA ablation of nNOS ACs, no response rectification was observed for either spot size (Figures S4A, S4G, and S4H). Similar to scotopic conditions, mesopic responses were also linear (Figures S4B, S4G, and S4H), suggesting that nNOS-1 presynaptic inhibition imparts negative rectification. Surround-evoked negative rectification was present in photopic AII responses (Figures S4C, S4G, and S4H), however, providing further evidence that nNOS-1 ACs do not contribute to surround suppression and rectification of AII output at the highest light levels. Taken together, these results suggest that some other spiking AC(s) contributes surround suppression to AIIs under photopic conditions.

nNOS-2 ACs, also ablated by DTA expression, may affect AII responses. nNOS-2 ACs release NO (nNOS1 ACs do not^59^), which modulates electrical coupling between HCs^60,61^ and between AIIs and ON CBCs.^62^ To examine HC contributions to the AII surround following nNOS AC ablation, we recorded AII EPSCs in these retinas. Under mesopic conditions, weak surround suppression was not altered significantly by GABA_C_ blockade (Figures S4I–S4K) but was eliminated by HEPES (Figure S4I–S4K), similar to results observed in WT (Figures 3D–3F). nNOS-2 ablation did not appear to affect AII-ON CBC synapses because similar photopic AII PSPs were recorded post-nNOS AC ablation compared to WT at 25,000 R*/rod/s (compare Figures 1G, 1H, 6F, and 6G), suggesting that excitatory synaptic transmission to AIIs was unperturbed.

### Changes in downstream encoding of photopic signals

Although we did not identify the AC(s) contributing to AII RF surrounds in photopic conditions, we observed a striking change in the time course of AII responses to large spot sizes at photopic backgrounds (Figure 1M). We found that mesopic surround inhibition simply scaled responses down without dramatic changes in kinetics. In photopic conditions, however, responses to wide-field sustained stimuli became brief: depolarization at light onset was followed closely by a transient hyperpolarization. We next tested whether this consequence of AII photopic surround inhibition is inherited by downstream circuitry.

**Figure 7.**
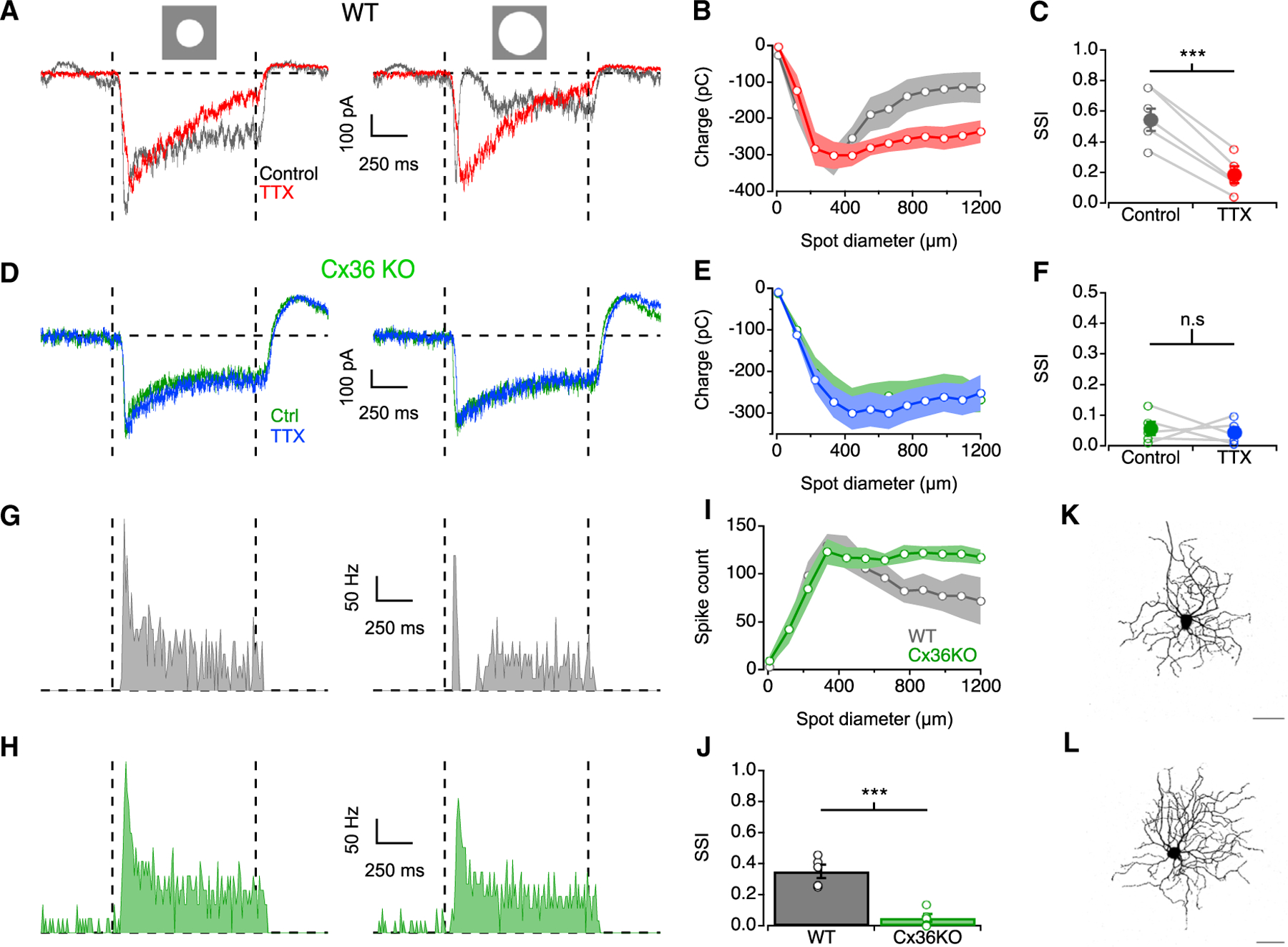
Changes in s-ONα responses at photopic levels (A) s-ONα EPSCs evoked by an 200 μm spot (left) and 1,200 μm spot (right) from a 25,000 R*/rod/s background (+100% Weber contrast). (B) Charge during stimulus interval vs. spot diameter at 25,000 R*/rod/s background (n = 5). (C) SSI plotted in control and TTX conditions across the cell population (open circles, individual cells; closed circles, population mean; n = 5). (D–F) As in (A)–(C) but in Cx36KO (n = 5). (G) WT s-ONα spike rate evoked by an 200 μm spot (left) and 1,200 μm spot (right) from a 25,000 R*/rod/s background (+100% Weber contrast). (H) As in (G) but in Cx36KO. (I) Spike count during stimulus interval vs. spot diameter measured at 25,000 R*/rod/s in WT and Cx36KO (n = 5). (J) SSI plotted in WT and Cx36KO mice across the cell population (open circles, individual cells; closed circles, population mean; n = 5 for both WT and Cx36KO). (K) Fluorescence micrograph of an Alexa 488-filled ONα RGC (max z-projection) in WT retina. Scale bar: 50 μm. (L) As in (K) but in Cx36KO retina. Data are represented as mean ± SEM. ***p < 0.001.

Kinetic changes in AII signaling observed here may significantly impact retinal output since AIIs are connected to most bipolar types that relay signals to RGCs (Figure 1B).^28,63^ s-ONα RGCs receive inputs mostly from type 6 and type 7 ON CBCs,^64^ both of which form gap junctions with AIIs.^28^ Like in AIIs, s-ONα EPSCs were diminished by surround inhibition under mesopic and photopic, but not scotopic, conditions (Figures S5 and S7). Transient inhibition of responses to photopic stimuli, similar to that observed in AIIs, was present in s-ONα EPSCs for large-spot stimuli at 25,000 R*/rod/s and were eliminated by bath application of TTX (Figure 7A). Moreover, surround suppression of s-ONα EPSCs was reduced significantly by TTX (mean SSIs = 0.54 ± 0.07 and 0.18 ± 0.05 in control and TTX, respectively, p = 6.25 × 10^−5^, paired t test; Figures 7A–7C). These features of s-ONα light responses appear to be inherited from AIIs, as both the kinetic changes and surround suppression of s-ONα EPSCs were absent in Cx36KO retinas in which AIIs are disconnected from ON CBCs (Figures 7D–7F). Analogous effects were observed in s-ONα spike responses to large spots: in WT retinas, a transient burst of spikes at light onset was followed by a pause and resumption of sustained firing (Figures 7G and S6). This resembled the complex modification observed in AII responses and s-ONα EPSCs and was quite distinct from the sustained firing of s-ONα RGCs evoked by small spots (Figure 7G). In Cx36KO mice, ONα RGCs exhibited sustained responses for all spot sizes, and we did not observe any change in firing patterns for larger stimuli (Figure 7H). Similar to EPSCs, we observed strong surround suppression in WT s-ONα spikes that was absent in Cx36KO retinas (mean SSIs = 0.35 ± 0.04 and 0.04 ± 0.02 in WT and Cx36KO, respectively, p = 0.0002, unpaired t test; Figures 7I and 7J). Although additional layers of inhibition likely contribute to the surround suppression observed in s-ONα RGCs,^65^ these results highlight that changes in upstream AII RFs are inherited by downstream circuit elements.

## DISCUSSION

AIIs are present in the retinas of all mammals studied (including humans)^66–68^ and constitute the most numerous AC type, at least in mouse.^69^ AIIs are extensively coupled to most CBC types via either electrical or chemical synapses and thereby facilitate integration and interaction between multiple parallel pathways^28,63^ that collectively underlie visual processing at all times of day. Here, we explored mechanisms that shape AII RFs and dissected contributions from multiple layers of interneuron processing. Our experimental conditions minimized changes in the RF center size due to gap junction modulation,^26,27^ permitting us to study luminance-dependent contributions of wide-field interneurons. Under our scotopic conditions, an inhibitory surround was largely absent from AII light responses. Increasing the mean luminance by three orders of magnitude to mesopic levels recruited a divisive presynaptic surround from wide-field nNOS-1 ACs and, to a lesser extent, from HCs in the outer retina. As luminance was further increased to photopic levels, a different, yet unidentified source of surround suppression produced a dramatic change in AII response kinetics that was inherited by downstream circuits, strongly suppressing s-ONα RGC spike responses to large, uniform stimuli. These results highlight the context-dependent nature of circuit recruitment within the retina and provide insights into the mechanisms underlying these changes.

### Impact on RGC computations

Electrophysiological and anatomical studies have shown that AIIs are synaptically connected to ~85% of CBCs.^28,39,70^ Interpreting our results in the context of AII-CBC connectivity makes predictions for which circuits will be affected most strongly by the changes observed here. AIIs are electrically coupled to all ON CBCs except 5b, with particularly strong connectivity with CBC types 5a, 6, and 7.^28^ Thus, RGCs that receive excitatory inputs from these cells, like the s-ONα, will likely be impacted by these surround effects. Indeed, under photopic conditions, excitatory synaptic inputs to the s-ONα exhibit the same kinetics and pharmacological profile observed in AIIs (Figure 7). s-ONα RGCs receive synaptic input from type 6 and type 7 CBCs,^64^ both of which are heavily connected to AIIs via gap junctions. Deleting Cx36 expressed by AIIs eliminated the signal transformation, indicating that AII connectivity with CBCs is required to provide the inhibitory surround in s-ONα photopic RFs. The presynaptic surround inhibition observed in AII photopic RFs must therefore impinge on a CBC that is electrically coupled to AIIs but provides little excitatory input to s-ONαs. The anatomical connectivity noted above^28^ suggests CBC 5a as the most likely candidate. Similarly, AIIs make many glycinergic synapses onto types 2, 1a, and 3b, and to a lesser extent onto types 3a and 4,^28,39^ suggesting that the AII’s inhibitory surround may shape OFF signaling as well. Together, these data predict that many known retinal circuits, e.g., direction selectivity, looming, suppressed by contrast, and local edge detection, may be influenced by AIIs. Although many bipolar cell types likely inherit features of AII RFs, inhibition from other ACs confers signaling diversity across bipolar cell types.^71^

Retinal adaptation was originally thought to invoke a simple gain control mechanism that gives rise to a luminance-independent retinal code.^72^ However, recent reports have shown that adaptation can extend far beyond simple gain control, leading some circuits to encode distinct information under different luminance conditions.^34,73,74^ Through pharmacology and genetic manipulation, we have identified luminance-dependent mechanisms shaping AII RFs that likely play a role in multiple dynamic response properties (e.g., sustained vs. transient, polarity preferences) evident in multielectrode array recordings.^73,74^ More work is needed to determine how signals from AIIs are integrated with CBCs to underlie these complex transformations at the level of retinal output.

The mechanisms presented here may relate to previously described size-selectivity computations performed by s-ONα circuits.^65^ This earlier study reported that wide-field, high-contrast flicker stimuli suppressed spike output when peak light levels reached ~10 R*/rod/s. The direct inhibition to RGCs observed under these conditions may combine with the presynaptic mechanisms shown here to reinforce this computation.

### Role of electrical synapses in AII RFs

The retina employs a variety of Cx proteins to form both hetero- and homotypic gap junctions,^75^ and Cx36 plays a particularly important role in dim light vision.^31^ Under scotopic conditions, Cx36-containing gap junctions allow rod signals to be relayed to the cone pathways via rod-cone gap junctions and AII-ON CBC gap junctions. A third set of Cx36-containing gap junctions mediate direct interactions between AIIs, increasing the effective collecting area of these cells.^24,38,76^ Studies in rabbit have shown that AII RFs expand under mesopic conditions due to modulation of the AII-AII gap junctions. We find in C57BL/6 mice that gap-junctional modulation of AII RFs is almost completely absent in our preparation, perhaps due to several reasons. First, dopamine, NO, and Cx36-containing gap junctions are modulated by adaptation that occurs over slower time scales^26,27,59^ (~45–60 min) compared to the duration of our recordings at each background luminance level (10–15 min). Moreover, in previous studies reporting light-induced modulation of AII-AII coupling,^26,27^ the eyecup was kept intact during the experiment. We suspect that our flat mount retina preparation, lacking the retinal pigment epithelium and potentially other regulatory elements, might diminish neuromodulation of electrical synapses. The high perfusion rate (8–9 mL/min) in our experiments ensures consistent light responses but is substantially faster than that in studies measuring light-induced dopamine release^77^ and limits dopamine’s modulatory effects. Although the whole-mount preparation carries these caveats, it has enabled us to examine the effects of light levels and synaptic inhibition on AII RFs independently of potentially confounding gap junction effects.

### Limitations of the study

Because our preparation, as mentioned above, minimizes modulation electrical coupling, further experiments are required to integrate these and other adaptation mechanisms with the circuit features described here to reach a complete understanding of AII RFs. The prevalence of surround inhibition, moreover, may depend on details of the light stimuli: here, in response to moderate spatial and temporal contrast (±100%) stimuli atop a scotopic (0.5 R*/rod/s) background, AII RFs lacked an inhibitory surround, whereas in previous work, stronger scotopic stimuli (~10 R*/rod/s) atop a completely dark background (i.e., theoretically infinite contrast) recruited surround inhibition.^42^ In addition, much stronger stimuli from darkness^42^ likely activate input via both rod and cone pathways, whereas our moderate contrast photopic stimuli, delivered atop a bright background, recruited only cone bipolar input (Figure 1; see STAR Methods). Although rods can signal at photopic light levels, the relatively brief time (5–10 min) spent at each light level in our experiments was insufficient to allow rods to recover sensitivity following a change in background luminance.^78^

Finally, while the results presented here indicate that one RGC type, the s-ONα, inherits its inhibitory surround from AIIs, further experiments are required to determine whether other RGCs also inherit RF characteristics from these versatile interneurons.

## STAR★METHODS

### RESOURCE AVAILABILITY

#### Lead contact

Further information and requests for resources should be directed to and will be fulfilled by the lead contact: Jeffrey Diamond, 35 Convent Dr., Building 35A, Room 3E-621, Bethesda, MD 20814 USA; diamondj@ninds.nih.gov.

#### Materials availability

This study did not generate new unique reagents.

#### Data and code availability

- Original electrophysiology data have been deposited at Mendeley and are publicly available as of the date of publication. The DOI is listed in the key resources table. Microscopy data reported in this paper will be shared by the lead contact upon request.
- This paper does not report original code.
- Any additional information required to reanalyze the data reported in this paper is available from the lead contact upon request.

### EXPERIMENTAL MODEL AND STUDY PARTICIPANT DETAILS

Mice of either sex between 4 and 25 weeks were dark adapted overnight. Experiments were performed from the following mouse lines: C57/BL6, Cx36^−/−^, nNOS-CreER/Ai14-TdTom. Animals were sacrificed according to NIH guidelines and were approved by the NINDS Animal Care and Use Committee (ASP 1344).

### METHOD DETAILS

#### Electrophysiology

Retinas were dissected under infrared illumination (940 nm LED, ThorLabs) with assistance from IR visible light converter (night vision) goggles and separate IR dissection scope attachments (BE Meyers). After removal from eye cup, 4 relieving cuts were made on the retina and placed flat onto a poly-D-lysine coated glass coverslip (12mm diameter, Corning BioCoat Cellware) that was secured to a recording dish via grease (Dow Corning) and a harp (ALA Scientific, HSG 5A) was put over the tissue. Retinas were mounted photoreceptor side down. Tissues were perfused with Ames medium (285mOsm, 7–9 mL/min) maintained at a temperature of 30-32°C. For AII recordings, a diagonal tunnel was burrowed from the GCL to the INL-IPL border using an electrode and a second electrode was used for recordings. ON α RGCs were identified by their large soma size. Cell attached and whole cell recordings were made with an electronic amplifier (MultiClamp 700B, Molecular Devices) and signals were collected at a sample rate of 10kHz. For voltage-clamp recordings, patch electrodes (1.5mm OD borosilicate glass, 3–4 MΩ for RGCs, 6–8 MΩ for AIIs) were filled with internal solution containing (in mM): 105 CsCH_3_SO_3_, 10 TEA-Cl, 20 HEPES, 10 EGTA, 1 MgCl_2_, 1 NaCl, 10 Phosphocreatine di(tris), 2 QX-314, 5 Mg-ATP, and 0.5 Na-GTP, 0.1 Alexa 488/Alexa 568 hydrazide at 265–270 mOsm, pH = 7.4 with CsOH. For current clamp recordings, patch electrodes were filled with internal solution containing (in mM): 123 KCH_3_SO_3_, 10 HEPES, 1 MgCl_2_, 1 NaCl, 2 EGTA, 7 Phosphocreatine di(tris), 4 Mg-ATP, and 0.5 Na-GTP, 0.1 Alexa 488/Alexa 594 hydrazide at 265–270 mOsm, pH = 7.4 with KOH. Absolute voltage values were corrected for a −8.58 mV liquid junction potential in the cesium-based intracellular solution. Concentrations of pharmacological agents were used as follows: TTX (500nM), HEPES (20mM), gabazine (10μM), strychnine (1μM), TPMPA (50μM), NBQX (10μM). Recordings were resumed 1–2 min after drug perfusion and 5 min after HEPES perfusion.

#### Visual stimuli

Light stimuli were presented using a customized 912 × 1140-pixel digital projector (DLPLCR4500; Texas Instruments) driven by a 405-nm LED (ThorLabs) at a frame rate of 60 Hz.^81^ Spatial stimuli patterns were created with MATLAB-based software (https://github.com/Schwartz-AlaLaurila-Labs/sa-labs-extension). Photon flux was attenuated to desired levels using a motorized neutral density filter wheel (FW102C, Thorlabs) and routed through the microscope (Scientifica Hyperscope) condenser, which was adjusted so that images were in focus at the plane of the photoreceptor outer segments. Photoisomerization rates were calculated based on a collecting area of 0.85 μm^2^ for rods.^82^ Responses to horizontal and vertical bars (50 μm × 500 μm) presented across 11 locations along each axis spaced by 20μm for AIIs and 40 μm for both sustained ONα and transient ONα (t-ONα) were measured to obtain the spatial position of receptive field (RF) center. Subsequent stimuli were delivered at the RF center. Circular spots of 100μm and 200μm diameter on dark background were used to identify light step profiles of AIIs and ON α RGCs respectively. In Figure 7, stimuli of +300% Weber contrast were used In Cx36KO retinas due to reduced sensitivity of RGCs in these mice.^31^ Spots of diameters ranging from 10 to 1200 μm were used to characterize RF architecture and center surround organization. Annuli of 250μm inner diameter and 1000μm outer diameter were used for stimulating the surround in Figures 4 and S3. Temporal frequency stimuli consisted of a 100μm or 1000μm spot whose contrast was modulated sinusoidally (0.5–20 Hz frequency range) between +100% and −100% from the mean background. All stimuli with varying parameters were presented in pseudorandom order. Cells were adapted to a background luminance for 50-100s^34^ after a change in mean luminance prior to data acquisition. Increasing mean luminance typically induced sustained firing similar to that reported previously in s-ONα RGCs,^83^ but this activity diminished in 30–60 s as the cell adapted to the new light level, resulting in low baseline firing rates (Figure S6B).

#### Virus injections

The single-stranded AAV vector, AAV2/7m8-CAG-FLEX-DTA-WPRE-SC40pA (>10^13^ viral genome particles/mL) was produced was intravitreally injected in nNOS-CreER/TdTom mice. Mice were anesthetized with isoflurane (2–3% at 1.5 L/min). Using a 30-gauge needle, a small hole was made at the margin of the cornea and sclera. The AAV preparation (~1.5 μL) was injected through this hole using a Hamilton injection system (syringe: 7633–01, needle: 7803–05, point style 3, length 10 mm). After injection, mice were returned to their home cage and monitored, until fully recovered. 2 days post intraocular injections, Cre expression was induced by intraperitoneal injection of tamoxifen dissolved in corn oil (20 mg/mL), administered in 3 doses (2mg tamoxifen each dose) over a period of 5 days.

#### Immunohistochemistry

Tissues were fixed for 30 min in 4% paraformaldehyde (Electron Microscopy Sciences) and incubated in 0.1 M phosphate buffer saline (PBS) overnight at 4°C. Fixed retinas were incubated in PBS containing 3% normal donkey serum (blocking agent), 0.05% sodium azide, 0.5% Triton X-100 for 2 h. This was followed by incubation in blocking solution and primary antibodies against nNOS (1:500 v/v) and ChAT (1:100 v/v) for 5 nights at 4°C. Afterward, tissues were rinsed in PBS and incubated for 2 nights at 4°C in blocking solution with secondary antibodies; donkey anti-rabbit (1:250 v/v) and donkey anti-goat (1:250 v/v). Retinas were then mounted on a slide using Vectashield Antifade medium (Vector Labs).

#### Imaging

After whole cell recordings, AII or ONα morphology was imaged using two photon microscopy (800 nm Chameleon laser) under a 20X water immersion objective (Olympus XLUMPlanFL N, NA 1.00) for cell identification. Fixed tissues were imaged on a Zeiss LSM 800 laser scanning confocal microscope equipped with a 40X oil immersion objective (Plan-Apochromat, NA 1.3). nNOS, TdTom and ChAT labeling were imaged at 488, 568 and 647 nm excitation, respectively. All confocal images were collected with spacing of 0.5 μm in the z axis.

#### Data analysis

Peak depolarizations were calculated during the stimulus period, averaged over 4 trails and then averaged over the population. For voltage clamp experiments, charge was calculated as current integrated over the stimulus time window averaged across 4 trials. Surround suppression index (SSI) was calculated as follows:

$$SSI=\frac{Resp_{max}-Resp_{1200\mu m}}{Resp_{max}+Resp_{1200\mu m}}$$

where Resp_max_ and Resp_1200μm_ are the maximum response across all spot sizes and response to a 1200μm spot respectively.

In Figures 4 and S4, rectification index (RI) was defined as:

$$RI=\frac{Resp_{+100\%}-Resp_{-100\%}}{Resp_{+100\%}+Resp_{-100\%}}$$

where Resp_+100%_ and Resp_−100%_ are the responses to +100% and −100% contrast respectively. The responses at +100% and −100% contrasts were always opposite in polarity.

In Figure 7, peristimulus time histograms were calculated using a sliding time window of 100ms.

All electrophysiological data were analyzed in MATLAB, using a custom written open-source package (http://www.github.com/SchwartzNU/SymphonyAnalysis). Figures were constructed in IgorPro 8.04 (Wavemetrics) and Adobe Illustrator 2022.

### QUANTIFICATION AND STATISTICAL ANALYSIS

Data are reported as mean ± SEM. P-values for comparisons were calculated using a two tailed Student’s T-Tests (paired or unpaired as appropriate) unless specified otherwise. One way ANOVA was used to calculate p values in Figures 5N–5Q. No statistical methods were used to predetermine sample sizes.

## Supplementary Material

1

## Figures and Tables

**Table:** KEY RESOURCES TABLE

REAGENT or RESOURCE	SOURCE	IDENTIFIER
**Antibodies**		

α-ChAT (goat polyclonal)	EMD Millipore	Cat# AB144P; RRID: AB_2079751
α-nNOS (rabbit polyclonal)	ThermoFischer Scientific	Cat# 61-7000; RRID: AB_2313734
α-goat (donkey) FITC	Jackson ImmunoResearch	Cat# 705-095-147; RRID: AB_2340401
α-rabbit (donkey) Cy5	Jackson ImmunoResearch	Cat# 705-095-147; RRID: AB_2340607

Bacterial and virus strains		

AAV2/7m8-CAG-FLEX-DTA-WPRE-SC40pA	Gift from Dr. Jonathan Demb (Park et al.)^42^	

Chemicals, peptides, and recombinant proteins		

Ames media	US Biological Life Sciences	A1372-25
NaHCO_3_	Fischer Bioreagents	BP328-500
CsCH_3_SO_3_	Millipore Sigma	C1426
KCH_3_SO_3_	Millipore Sigma	83000
TEA-Cl	Millipore Sigma	T2265
Mg ATP	Millipore Sigma	A9187
Na GTP	Millipore Sigma	G8877
EGTA	Millipore Sigma	E8145
Phosphocreatine di(tris)	Millipore Sigma	P1937
HEPES	Millipore Sigma	H3375
NaCl	Millipore Sigma	S9888
MgCl_2_	Millipore Sigma	63069
QX-314	Alomone Labs	Q-100
Alexa Fluor 488	ThermoFischer	A10436
Alexa Fluor 594	ThermoFischer	A10438
NBQX	Tocris Bioscience	0373
TTX	Alomone Labs	T-550
SR-95531	Tocris Bioscience	1262
Strychnine	Millipore Sigma	S8753
TPMPA	Tocris Bioscience	1040

Deposited data		

Igor Pro experiment files containing electrophysiological data and micrographs	Mendeley	https://doi.org/10.17632/2f22z599ww.1

Experimental models: Organisms/strains		

mouse: wild-type: C57BL/6J	Jackson Laboratory	RRID:IMSR_JAX:000664
mouse: nNOS-CreER	Jackson Laboratory	RRID:IMSR_JAX:014541
mouse: Ai14	Jackson Laboratory	RRID:IMSR_JAX:007914
mouse: Cx36^−/−^	Gift from Dr. David Paul (Deans et al.)^31^	RRID: MGI:3810172

Software and algorithms		

Stage	Github	https://github.com/Stage-VSS/stage
Symphony	Github	https://github.com/Symphony-DAS/symphony-matlab
Data Analysis package	Github	https://github.com/Schwartz-AlaLaurila-Labs/sa-labs-extension
MATLAB	Mathworks	RRID: SCR_01622
Igor Pro	Wavemetrics	https://www.wavemetrics.com/products/igorpro
Adobe Illustrator	Adobe	https://www.adobe.com/products/illustrator.html
Fiji	ImageJ	https://ImageJ.net/software/fiji/

## Highlights

- AII (“A-two”) amacrine cell (AC) receptive fields (RFs) change with increasing luminance
- Surround inhibition, absent in scotopic (dim light) AII RFs, emerges at higher light levels
- nNOS1 ACs provide inhibitory surround to AIIs under mesopic, but not photopic, conditions
- Inhibitory surround observed in some downstream ganglion cell RFs is inherited from AIIs
