# Supplementary material for: Layers of inhibitory networks shape receptive field properties of AII amacrine cells
Source: Cell Rep. Author manuscript; Available in PMC 2024 Jan 5. (PMC10769003; doi:10.1016/j.celrep.2023.113390)
Supplement: 1 [file NIHMS1948167-supplement-1.pdf]

Cell Reports, Volume *42*

Supplemental information

Layers of inhibitory networks shape receptive field properties of AII amacrine cells

Amurta Nath, William N. Grimes, and Jeffrey S. Diamond

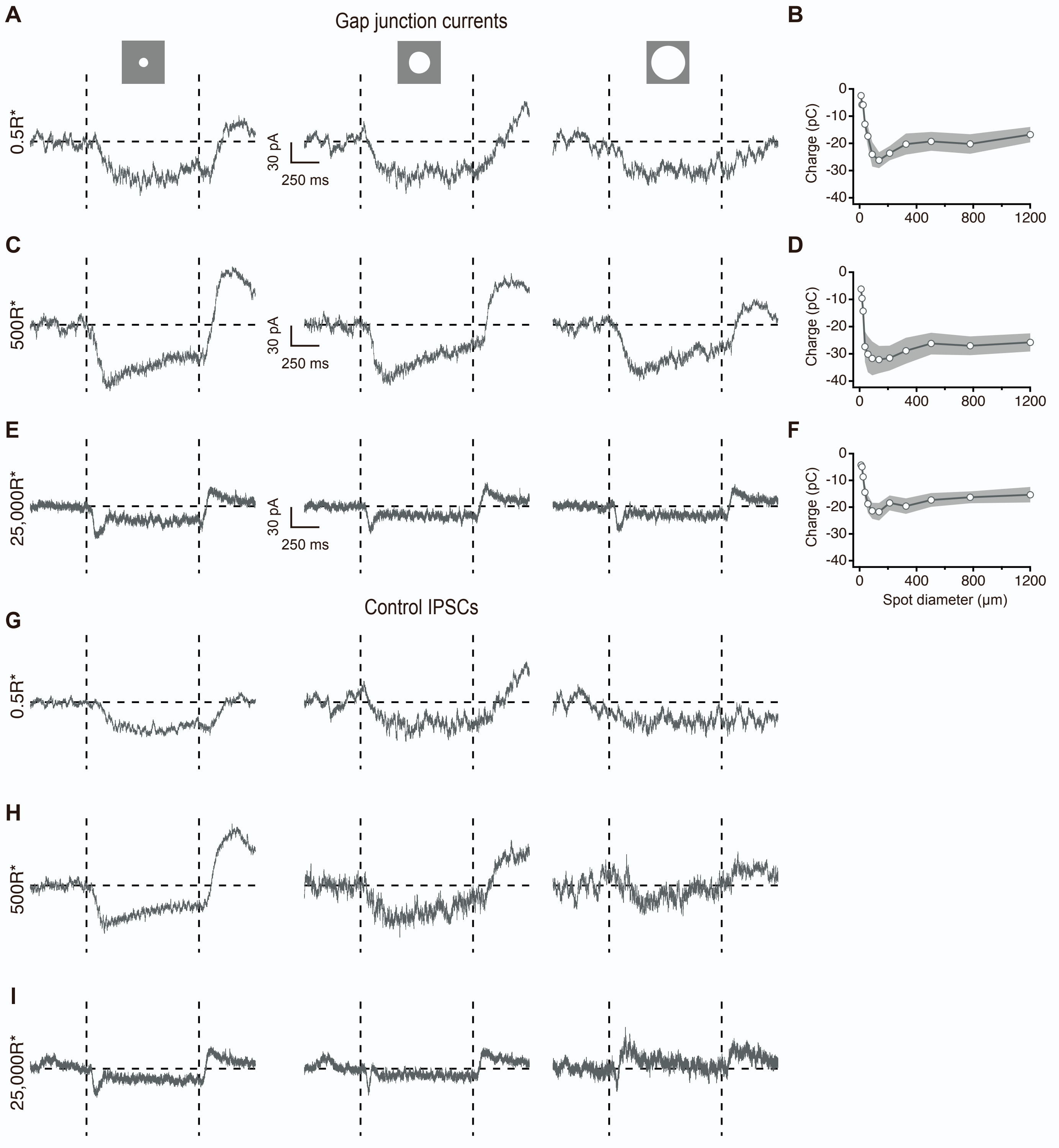

Figure S1. Gap junctional inputs to AII under different background luminance (Related to Figure 2)
(A) AII IPSCs measured after bath administration of TTX to a 88µm spot (*left*), 325µm spot (middle) and 1200µm spot (*right*) of +100% Weber contrast from a 0.5R*/rod/s background.
(B) Charge during stimulus interval versus spot diameter measured at 0.5R*/rod/s background. Shaded regions represent ±SEM across cells (n=8).
(C-D) As in (A-B), 500R*/rod/s background (n=8).
(E-F) As in (A-B), 25,000R*/rod/s background (n=8).
(G) AII IPSCs measured in control conditions to a 88µm spot (*left*), 325µm spot (middle) and 1200µm spot (*right*) of +100% Weber contrast from a 0.5R*/rod/s background.
(H) As in (G), 500R*/rod/s background.
(I) As in (G), 25,000R*/rod/s background.

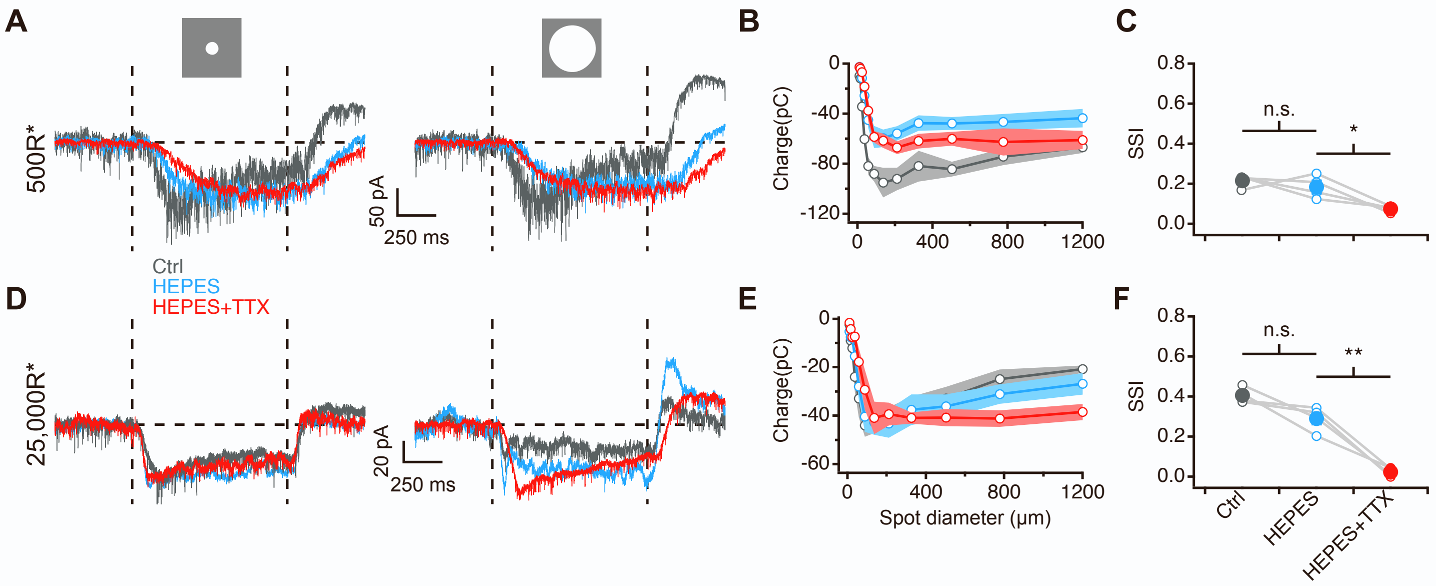

Figure S2. Contribution of horizontal cells to presynaptic surround suppression (Related to Figure 3)

(A) AII EPSCs in response to an 88µm spot (*left*) and 1200µm spot (*right*) of +100% Weber contrast from a 500R*/rod/s background.

(B) Charge during stimulus interval versus spot diameter measured at 500R*/rod/s background (n=4).

(C) SSI plotted in control, HEPES and HEPES+TTX across the population of cells (open circles, individual cells; closed circles, population mean, n=4).

(D-F) As in (A-C), 25,000R*/rod/s background (n=4). Data are represented as mean ± SEM.

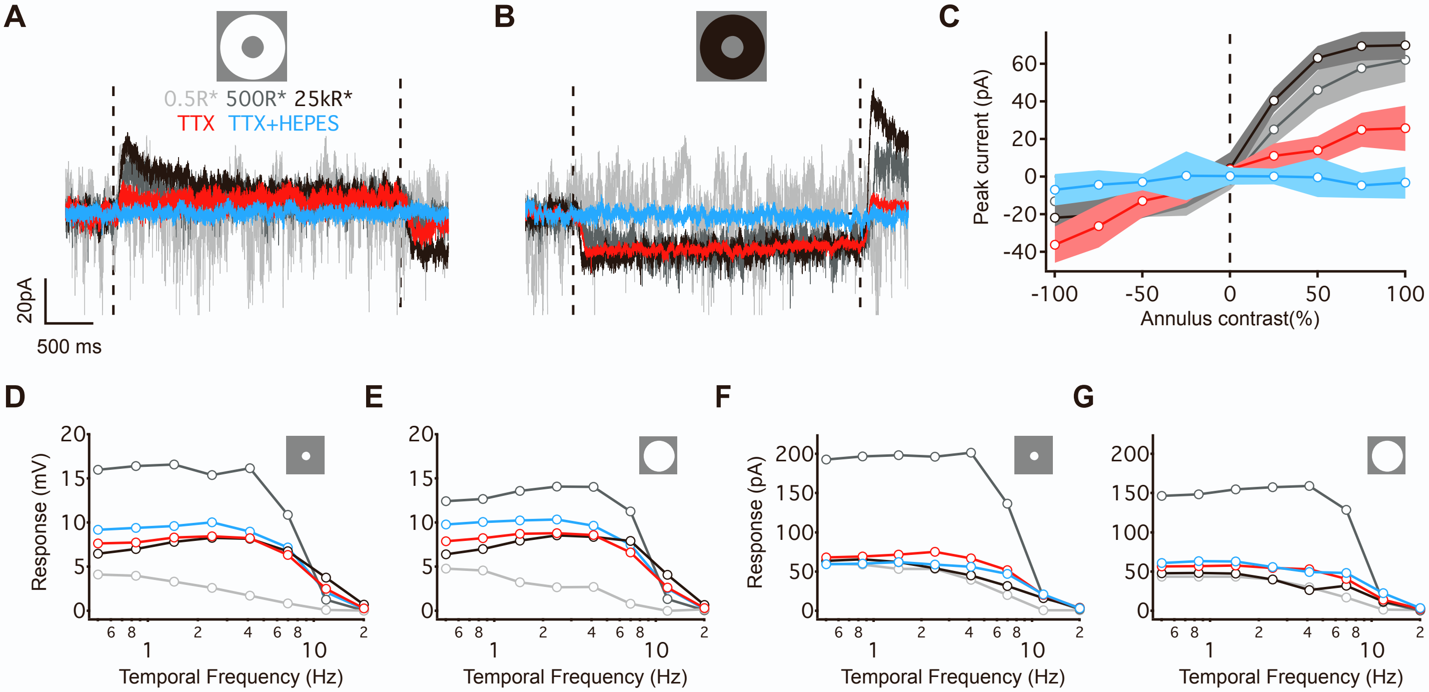

Figure S3. Presynaptic inhibition rectifies AII excitatory input (Related to Figure 5)

(A) AII EPSCs AII evoked by an annulus (100µm inner diameter, 1000µm outer diameter, +100% Weber contrast) at different backgrounds.

(B) As in (A), for -100% Weber contrast.

(C) Summary of AII membrane depolarizations versus annulus contrast (n=5 cells for 500R*/rod/s, 25,000R*/rod/s and TTX conditions, n=4 cells for TTX+HEPES condition).

(D-E) Summary of AII membrane potential responses to a temporally modulated 100 µm spot (D) and a 1000 µm spot (E) for the example cell shown in (Figures 5D-5H).

(F-G) Summary of AII EPSCs to a temporally modulated 100 µm spot (F) and a 1000 µm spot (G) for the example cell shown in (Figures 5I-5M). Data are represented as mean ± SEM.

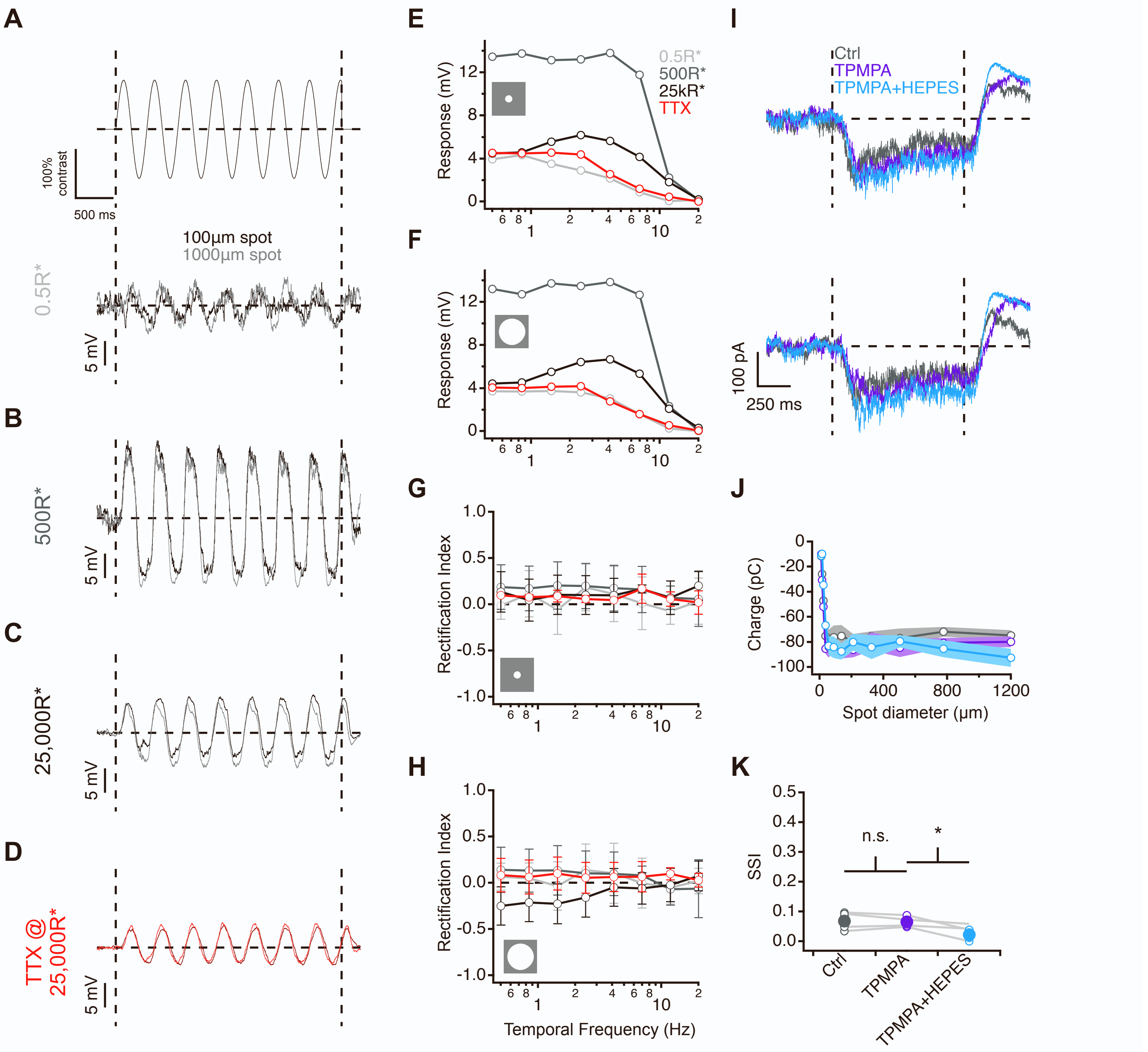

Figure S4. Ablating nNOS1 removes AII response rectification (Related to Figure 6)

(A) AII membrane responses (bottom row) to stimuli with sinusoidally modulated contrast (top row) in DTA ablated nNOS-CreER/TdTom retina. Responses to 100 µm spot (black) and 1000 µm spot (dark gray) are plotted together for a 2.43Hz temporally modulated stimulus presented from a mean background luminance of 0.5R*/rod/s.

(B-D) As in (A) but for background of 500R*/rod/s (B), 25,000R*/rod/s (C), TTX perfused at 25,000R*/rod/s (D).

(E-F) Summary of AII membrane potential responses to a temporally modulated 100 µm spot (E) and a 1000 µm spot (F) for the example cell shown in (B-D).

(G-H) Rectification index of AII membrane responses plotted versus temporal frequency (n=6).

(I) AII EPSCs in response to an 88µm spot (top) and 1200µm spot (bottom) of +100% Weber contrast from a 500R*/rod/s background.

(J) Charge during stimulus interval versus spot diameter at 500R*/rod/s background (n=4).

(K) SSI plotted in control, TPMPA and TPMPA+HEPES conditions across the population of cells (open circles, individual cells; closed circles, population mean, n=4). Data are represented as mean ± SEM.

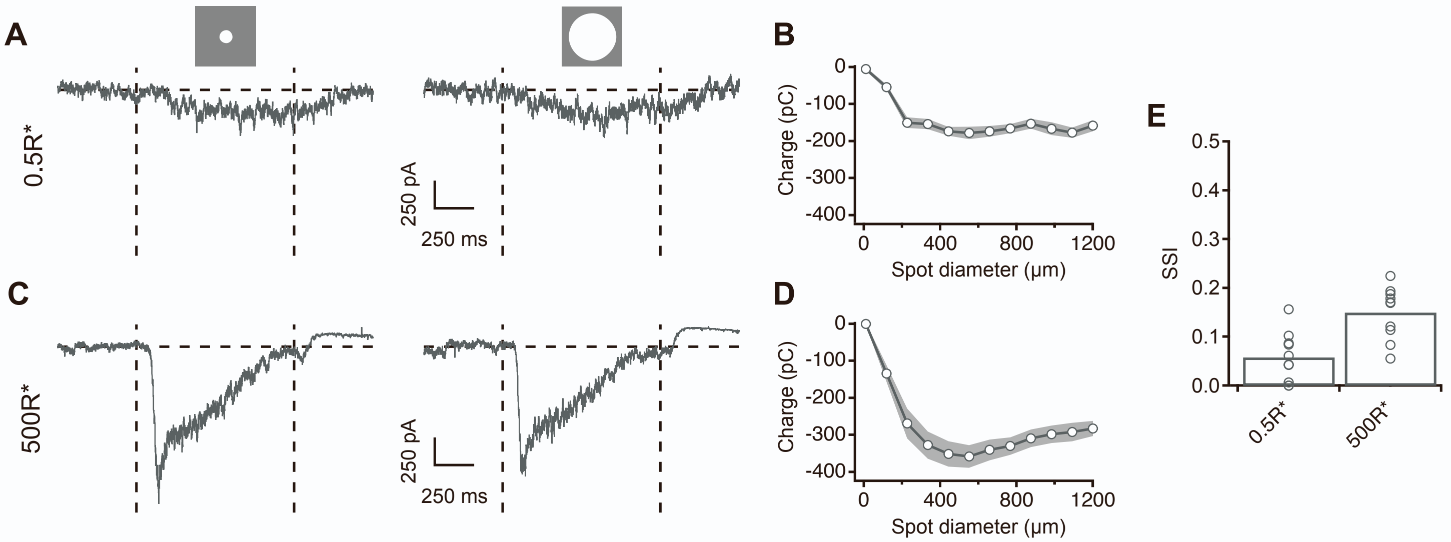

Figure S5. Scotopic and mesopic RFs of s-ONα RGCs (Related to Figure 7)
(A) s-ONα EPSCs evoked by an 200μm spot (*left*) and 1200μm spot (*right*) from a 500R*/rod/s background (+100% Weber contrast).
(B) Charge during stimulus interval versus spot diameter at 500R*/rod/s background (n=10).
(C-D) As in (A-B), 25,000R*/rod/s background (n=10).
(E) SSI plotted at 0.5R*/rod/s and 500R*/rod/s backgrounds across the cell population (open circles, individual cells; n=10 both conditions).

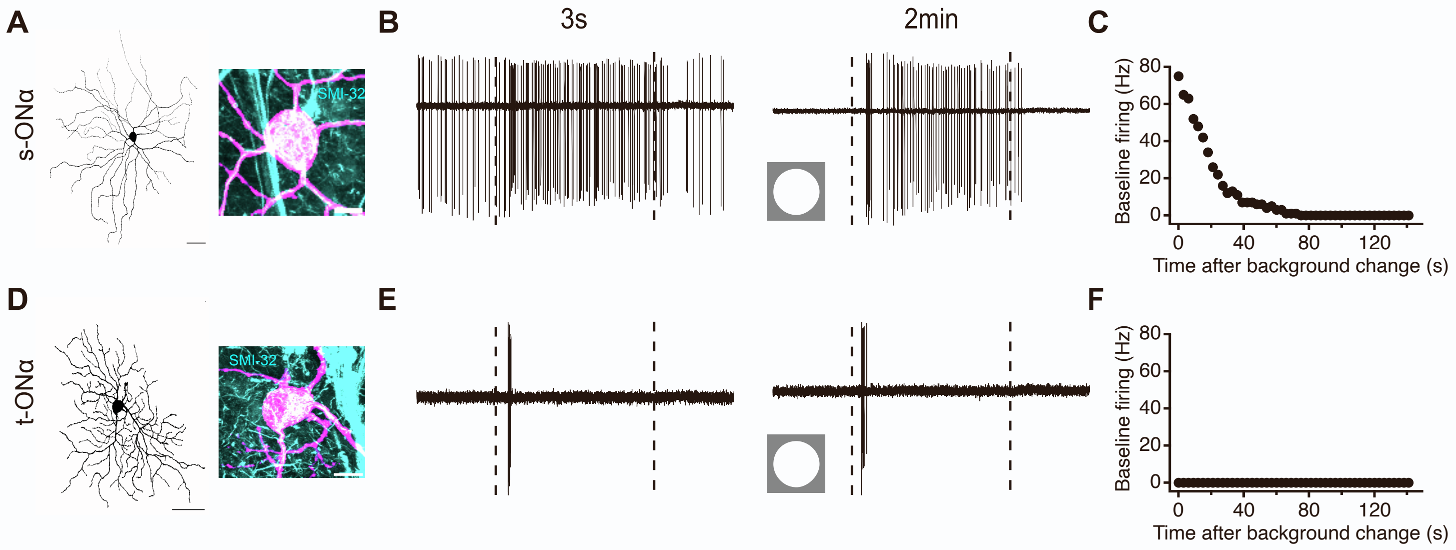

Figure S6. Identification and baseline firing of ONα RGCs (Related to Figure 7)

(A) *Left*, Fluorescence micrograph of an neurobiotin filled s-ONα RGC (max z-projection) in WT retina. Scale bar = 50 µm. *Right*, the same cell (magenta) expressed SMI-32 (cyan), a marker for αRGCs. Scale bar = 10 µm.

(B) Cell-attached recordings of light responses evoked responses 3 s after applying a 25,000R*/rod/s background (*left*) and 2 minutes later (*right*).

(C) Diary plot showing that the baseline firing rate recorded in the s-ONα RGC decreased dramatically during the first 80 seconds after changing to a higher background luminance.

(D-F) As in (A-C), but for a transient ONα RGC (Krieger et al., 2017), in which no baseline firing was observed at any time during the experiment.
